# Gut microbiome modulation by *Veillonella ratti* induces resistance to EAE pathogenesis via microbe-derived metabolites

**DOI:** 10.1038/s12276-026-01779-z

**Published:** 2026-07-14

**Authors:** Panida Sittipo, Joon-Young Park, Eunike Tiffany, Ara Oh, Subin Moon, Chan Hee Lee, Jae Sang Oh, Tae-Young Kim, Mi-Na Kweon, Jaeseoung Choi, Keon-Hyoung Song, Dong-Woo Lee, Myung Hee Nam, Soo-Jong Hong, Eun-Young Lee, Seong Ran Jeon, Ho-Yeon Song, Bong-Soo Kim, Yun Kyung Lee

**Affiliations:** 1 Department of Integrated Biomedical Science, Soonchunhyang Institute of Medi-Bio Science, Soonchunhyang University, Cheonan, Republic of Korea; 2 Department of Pharmacology and Biopharmaceutical Sciences, Faculty of Pharmaceutical Sciences, Burapha University, Chon Buri, Thailand; 3 Department of Life Science, Multidisciplinary Genome Institute, Hallym University, Chuncheon, Republic of Korea; 4 Department of Biomedical Science, Hallym University, Chuncheon, Republic of Korea; 5 Department of Neurosurgery, Uijeongbu St Mary’s Hospital, College of Medicine, The Catholic University of Korea, Seoul, Republic of Korea; 6 Department of Medical Sciences, College of Medicine, The Catholic University of Korea, Seoul, Republic of Korea; 7 Mucosal Immunology Laboratory, Department of Convergence Medicine, University of Ulsan College of Medicine/Asan Medical Center, Seoul, Republic of Korea; 8 Department of Pharmaceutical Engineering, College of Medical Sciences, Soonchunhyang University, Asan, Republic of Korea; 9 Department of Biotechnology, Yonsei University, Seoul, Republic of Korea; 10 Metropolitan Seoul Center, Korea Basic Science Institute (KBSI), Seoul, Republic of Korea; 11 Department of Pediatrics, Humidifier Disinfectant Health Center, National Medical Center, Seoul, Republic of Korea; 12 Department of Biochemistry, College of Medicine, Soonchunhyang University, Cheonan, Republic of Korea; 13 Digestive Disease Center, Institute for Digestive Research, Soonchunhyang University College of Medicine, Seoul, Republic of Korea; 14 Department of Microbiology and Immunology, School of Medicine, Soonchunhyang University, Cheonan, Republic of Korea; 15 Department of Nutritional Science and Food Management, Ewha Womans University, Seoul, Republic of Korea

**Keywords:** Multiple sclerosis, Autoimmunity

## Abstract

The progression of multiple sclerosis (MS) is potentially influenced by the microbiome. Elucidating host–microbiome interactions in MS may aid in developing microbiome-based applications; however, these interactions remain unclear. Here, we aimed to elucidate how *Veillonella ratti* MHL0042, isolated from human infant feces, modulates neuroinflammation and disease severity in experimental autoimmune encephalomyelitis, a murine MS model. Whole metagenomic sequencing revealed that *V. ratti* MHL0042 reshaped disrupted gut microbiota via microbial interactions throughout the intestinal tract. *V. ratti* MHL0042 administration significantly reduced central nervous system inflammation, notably decreasing CD4^+^IFN-γ⁺ T cell populations and activated spinal cord microglia. Mechanistically, *V. ratti* MHL0042 depleted *pldA*-containing bacteria, involved in phosphatidylethanolamine metabolism, thus elevating dioleoyl phosphatidylethanolamine (DOPE) levels. Increased DOPE was not only detected in the intestinal tract but also extended systemically and reflected in the central nervous system. Exogenous DOPE administration recapitulated the attenuation of experimental autoimmune encephalomyelitis pathogenesis by suppressing microglial activation. These findings highlight the therapeutic applicability of the microbiome and underscore its potential in human disease treatment.

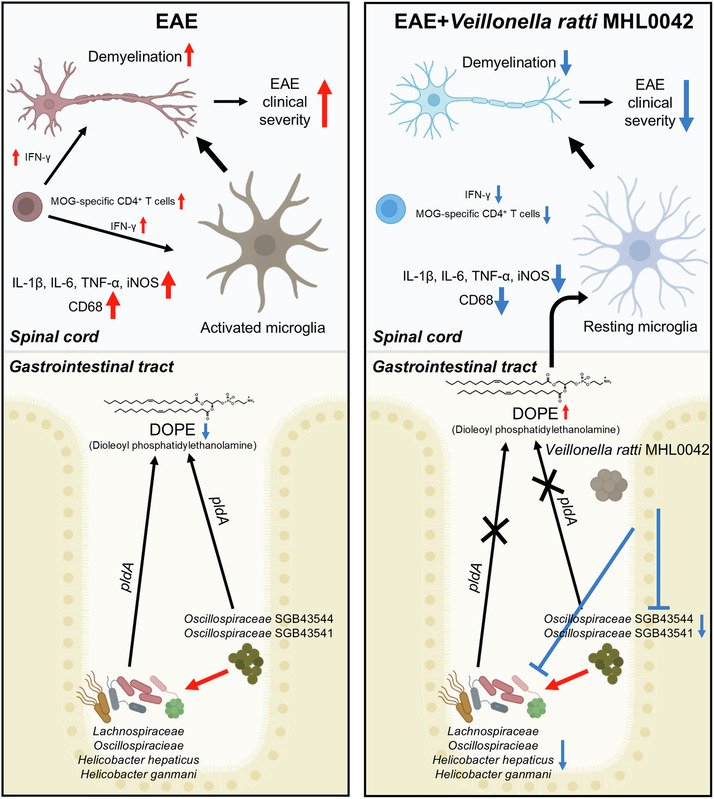

## Gut microorganism Veillonella ratti reduces MS disease severity

Multiple sclerosis (MS) is a chronic autoimmune disorder affecting the central nervous system, characterized by demyelination and neuroinflammation. This study explores the role of the gut microbiome, specifically the bacterium *Veillonella ratti*, in modulating MS pathogenesis using an experimental autoimmune encephalomyelitis (EAE) model. Researchers administered *V. ratti* MHL0042 to mice, observing significant reductions in EAE severity and demyelination. The study used whole metagenomic sequencing and metabolomic profiling to analyze changes in gut microbiota and metabolites. Results showed that *V. ratti* MHL0042 modulated the gut microbiota, particularly in the cecum, and increased levels of the metabolite dioleoyl phosphatidylethanolamine, which correlated with reduced neuroinflammation and microglial activation. These findings suggest that microbial composition changes by *V. ratti* MHL0042 and microorganism-derived metabolites, such as dioleoyl phosphatidylethanolamine, could offer new therapeutic avenues for MS by modulating the gut–central nervous system axis. IFN-γ, interferon-γ; iNOS, inducible nitric oxide synthase; MOG, myelin oligodendrocyte glycoprotein; TNF, tumor necrosis factor. "This summary was initially drafted using artificial intelligence, then revised and fact-checked by the author."

## Introduction

Multiple sclerosis (MS) is a chronic autoimmune disorder of the central nervous system (CNS) characterized by demyelination, neuroinflammation, and progressive neuronal degeneration. MS is caused by a complex interplay of genetic and environmental factors. Genome-wide association studies have identified several immune-related gene polymorphisms associated with MS susceptibility^1^. Autoreactive CD4⁺ T helper (T_H_) cells, particularly pathogenic T_H_1 and T_H_17 subsets, are key drivers of CNS inflammation, facilitating peripheral immune cell infiltration across the blood–brain barrier and triggering responses against CNS self-antigens^2^. In addition to T cells, microglia, which are tissue-resident macrophages of the CNS, have emerged as crucial regulators of neuroinflammation and disease progression in MS^3–5^.

The experimental autoimmune encephalomyelitis (EAE) model recapitulates many immunopathological features of MS and serves as an animal model to investigate disease mechanisms. EAE is induced by immunization with CNS antigens such as myelin oligodendrocyte glycoprotein (MOG), resulting in T cell-mediated CNS inflammation. Microglia not only respond to immune insults but actively contribute to disease by promoting antigen presentation, cytokine production, and demyelination^5–7^. Ablation or inhibition of microglia delays disease onset and reduces neuroinflammation, thus highlighting their pivotal role in EAE^8,9^.

Progressive evidence supports the role of the gut microbiome in modulating MS and EAE pathogenesis^10–12^. The gut harbors a complex microbial ecosystem which communicates with the immune system and influences both local and systemic immunity^13^. Patients with MS exhibit dysbiosis or altered gut microbiota, which is associated with increased abundance of pro-inflammatory taxa and reduced levels of immunoregulatory species^14,15^. Transferring microbiota from patients with MS into germ-free mice exacerbates EAE symptoms, whereas microbiota from healthy controls (HCs) exhibit protective effects^14^. Microbial metabolites, such as short-chain fatty acids and tryptophan derivatives, modulate T cell responses, microglial maturation, and neuroinflammation^16–18^. In particular, microglia exhibit profound functional alterations in the absence of microbial signals, underscoring the importance of gut microbial metabolites in brain immune crosstalk^16,17^.

Understanding how specific gut microbial taxa and their metabolites influence CNS inflammation is essential for developing microbiome-based therapies^19^. Notably, fecal microbiota belonging to the family Veillonellaceae are less abundant in patients with relapsing–remitting MS than that in HCs^20^. Recent microbiome profiling studies have identified *Veillonella* spp. as potentially immunomodulatory members of early-life and adult gut microbiome^21,22^. *Veillonella ratti* is an obligate anaerobic human intestinal bacterium, which ferments lactate into short-chain fatty acids, primarily acetate and propionate^23,24^. *V. ratti* can potentially modulate the gut microbiome and enhance host immune responses^25^. Coadministration of *V. ratti* with *Lactobacillus acidophilus* increases the abundance of *Akkermansia*, while decreasing the levels of potentially pathogenic genera such as *Escherichia–Shigella* and *Desulfovibrio*. This microbial alteration is associated with the reduced expression of pro-inflammatory cytokines, including IL-6, IL-1β, IL-17A, inducible nitric oxide synthase, and interferon (IFN)-γ, as well as attenuated oxidative stress. Thus, *V. ratti* may contribute to immune regulation and is a potential candidate for microbiome-based interventions in immune-mediated diseases such as MS.

In this study, we aimed to elucidate how human-derived *V. ratti* MHL0042 modulates neuroinflammation and disease severity in EAE. To achieve this, we followed an integrative approach combining whole metagenomic sequencing, analysis of immune cell responses, and assessment of microglial activation in the CNS, along with non-targeted metabolomic profiling. Our findings reveal a novel mechanism in which gut microorganism-derived metabolites, resulting from reconstruction of the gut microbiome by *V. ratti* MHL0042, suppress CNS inflammation and suggest the potential of microbiome-based strategies for treating MS.

## Materials and methods

### Public MS gut microbiome cohorts and sequence processing

To evaluate whether *Veillonella* sp. is consistently depleted in the gut microbiome of patients with MS, we analyzed publicly available datasets from independent cohorts comprising both whole metagenome shotgun (WMS) and 16S rRNA gene amplicon sequencing. The WMS dataset included the iMSMS cohort, whereas 16S amplicon datasets were obtained from Italian (PRJNA684124)^26^ and Chinese (PRJNA628832) cohorts^27^. Considering the differences in taxonomic resolution across study types, cross-cohort comparisons were primarily preformed at the genus level.

For the iMSMS cohort, processed metagenomic outputs (taxonomic profiling and functional annotation tables) were obtained from Dryad (DOI 10.7272/Q60C4T26)^28^. For the Italian and Chinese cohorts, Illumina-based 16S rRNA gene amplicon reads were retrieved from the Sequence Read Archive and processed using QIIME2 (v.2021.8.0)^29^. Quality filtering, denoising, paired-end read joining, and chimera removal were performed with the q2-dada2 plugin to generate amplicon sequence variants^30^. Taxonomy was assigned using a BLAST-based classifier (QIIME2 q2-feature-classifier) against the EzTaxon-e reference database^31^.

### *V. ratti* MHL0042 culture

*V. ratti* MHL0042 was isolated from infant feces^32^ using BACTO™ Brain Heart Infusion (BHI) medium (BD, Franklin Lake, NJ, USA) supplemented with 5 mg/l hemin (Sigma-Aldrich, St Louis, MO, USA). The isolated strain was subcultured on BHI agar for pure culture, and its taxonomic classification was evaluated using 16S rRNA sequencing. For animal model application, the identified strain was cultured using BHI broth medium at 37 °C for 16 h in an anaerobic workstation (Don Whitley Scientific Limited, Shipley, UK).

### EAE induction and *V. ratti* MHL0042 and dioleoyl phosphatidylethanolamine administration

Nine-week-old C57BL/6L female mice were purchased from ORIENT Bio (Korea) and were maintained in a specific pathogen-free animal facility at the Soonchunhyang Institute of Medi-Bio Science. EAE was induced using the Hooke Kit™ (EK-2110, Hooke Laboratories, Lawrence, MA, USA) containing materials per mouse for a single subcutaneous challenge with 200 µg MOG_35–55_ peptide emulsified in 200 µl of Complete Freund’s Adjuvant. On the day of immunization and 24 h post-challenge, mice were injected intraperitoneally with Pertussis toxin (200 ng per mouse; List Biological Laboratories; provided with the Hooke Kit). In the bacteria-treated group, mice were orally administered *V. ratti* MHL0042 (1 × 10^8^ CFU in 100 µl phosphate-buffered saline (PBS)) daily for a week before EAE induction and followed until the end of the experiment. At the indicated time points, contents of the proximal and distal small intestine, cecum, and colon, along with fecal samples, were collected for metagenomic sequencing. Immune profiling and histological analyses were performed at the peak of disease severity.

In the metabolite-treated group, mice were intraperitoneally injected with 100 µl of 25 μM dioleoyl phosphatidylethanolamine (DOPE; MedChemExpress, Monmouth Junction, NJ, USA) dissolved in 10% ethanol, 40% polyethylene glycol 300 (MedChemExpress), 5% Tween-80 (Sigma-Aldrich), and 45% PBS or orally gavaged with 250 μM DOPE dissolved in sodium carboxymethyl cellulose (Sigma-Aldrich) for 5 days before the EAE injection. The administration continued daily until disease onset and was followed by continued injection every alternate day until the peak of clinical symptoms. At the end of the experiments, the spinal cords were collected for histological analysis, immunofluorescence staining, and immune profiling. The protocol for this study was approved by the Institutional Animal Care and Use Committee of Soonchunhyang University (SCH24-0035).

### EAE evaluation

Mice were examined and scored daily for disease progression, as described previously^33^, using the following scoring: 0, clinically normal; 0.5, limp tail tip (when picked up by the base of the tail, the tail is tensioned, except for the tip); 1, limp tail; 1.5, limp tail and slight hind limb impairment with mildly unsteady gait; 2, limp tail, hind limb weakness, unsteady gait, and poor balance; 2.5, limp tail with hind limbs dragging and poor balance; 3, limp tail with complete hind limb paralysis, or limp tail with paralysis of one front leg and one hind leg; 3.5, limp tail and complete hind limb paralysis, with the mouse unable to sit right by itself when placed on its side; 4, limp tail, complete hind limb paralysis, and partial front limb paralysis (mouse is minimally mobile but appears alert and feeding); 4.5, limp tail, complete hind limb paralysis, and severe front limb paralysis (mouse is minimally mobile); and 5, complete paralysis. Disease progression was monitored daily and expressed as both mean clinical and cumulative clinical score. The cumulative clinical score was calculated as the sum of daily clinical scores for each mouse over the EAE period and reported as the group average with standard deviation.

### DNA extraction and whole metagenomic sequencing

Metagenomic DNA was extracted from each sample (proximal small intestine, distal small intestine, cecum, colon, and feces) using the RNeasy PowerMicrobiome Kit (Qiagen, Germantown, MD, USA), following the manufacturer’s instructions. A metagenomic library was prepared using the Swift 2S Turbo DNA Library Kit (Swift Biosciences, Ann Arbor, MI, USA), according to the manufacturer’s instructions. Purification and size selection were performed using HiAccuBead (AccuGene, San Diego, CA, USA), after the ligation step. Index PCR was performed using the Swift 2S Turbo Combinatorial Dual Indexing Primer Kit (Swift Biosciences), and purification and size selection were repeated using HiAccuBead. The library concentration was measured using Qubit™ dsDNA HS Assay Kit (Thermo Fisher Scientific, Waltham, MA, USA). Equimolar concentrations of each library sample were pooled to 4 nM and sequenced using the Illumina NovaSeq 6000 system (250-bp paired ends).

### Quantification of total bacterial content

The relative bacterial content in each sample was estimated and compared using quantitative real-time PCR based on 16S rRNA. Amplification was performed with the primers 340F (5′-TCCTACGGGAGGCAGCAG-3′) and 518R (5′-ATTACCGCGGCTGCTGG-3′) using the PCR Thermal Cycler Dice Real Time System III (Takara Bio, Shiga, Japan). Each sample was measured in triplicate with a final volume of 25 µl, containing 12.5 µl of 2× TB Green Premix EX Taq (Tli RNaseH Plus; Takara Bio), 2 µM of each primer, and 2 µl of DNA template (a tenfold dilution series of sample DNA) or distilled water (negative control). The amplification conditions were as follows: initial denaturation at 95 °C for 30 s; 40 cycles of denaturation at 95 °C for 5 s and annealing at 60 °C for 30 s, with a final extension at 72 °C for 10 min. The bacterial amounts were calculated by comparing threshold cycles values with a standard curve generated from parallel reactions of serial dilutions (1 × 10^1^– 1 × 10^7^) of 16S rRNA from the *Escherichia coli* K12 w3110 strain. The regression coefficient (*r*^2^) for all standard curves was ≥0.99.

### Whole metagenomic sequence processing

Trimmomatic was used to perform adapter trimming and quality filtering for raw sequence data^34^. PEAR (v.0.9.11) was used to merge paired-end sequences^35^. Contaminated mouse genes in the whole metagenomic sequence were aligned to the reference mouse genome GRCm39 using Bowtie2 (v.2.2.4)^36^ and removed using SAMtools (v.1.3.1)^37^. Taxonomic features were obtained based on species-level genome bins using MetaPhlAn4 (ref. ^38^). Functional features were obtained using Diamond against the Uniref90 database^39,40^. The resultant Uniref90 IDs were converted to Kyoto Encyclopedia of Genes and Genomes (KEGG) Orthologs^41^, the reads per kilobase of which were normalized using the cumulative sum scaling method.

### Reconstruction of metagenome-assembled genomes

Contigs were assembled using MEGAHIT (v.1.1.3)^42^. Binning was performed using metaBAT2, MaxBin2, and CONCOCT, followed by bin refinement using the metaWRAP (v.1.3.2) package^43–46^. The quality of each bin was verified using CheckM (v.1.0.12)^47^. Metagenome-assembled genomes (MAGs) with completeness > 50% and redundancy (contamination) < 10% were used for further analysis. Each MAG was taxonomically classified using GTDB-Tk (v.1.7.0)^48^. Pan-genome analysis was performed on MAGs classified as Lachnospiraceae, Oscillospiraceae, and *Helicobacter hepaticus* using Anvi’o (v.7)^49^. The KEGG Orthology gene families of each MAG were annotated using the “anvi-run-kegg-kofams” function.

### Microbiome analysis

Potential contaminants in the sequencing data were removed using the R package “decontam” based on the negative control sequences^50^. Decontamination was performed with a threshold of 0.5 using prevalence methods in “*isContaminant*” function. The taxonomic read counts were normalized by relative abundance for comparison between samples. Alpha diversity was calculated using the “*alpha*” function within the R package “microbiome”^51^. The microbiota was statistically analyzed using the R software (v.4.1.1)^52^.

The indicator species for each organ was identified based on the control group using the “*multipatt*” function of the R package “indicspecies” with 999 permutation^53^. Indicator analysis is a statistical method based on the relative abundance and frequency of species to determine whether a particular species is significantly more abundant in a defined group than that in other groups. The significance of the indicator was tested using 10,000 permutations. Species with results for *P* < 0.05 were considered as indicators for each organ in the gastrointestinal (GI) tract.

Significant differences among groups were analyzed using multivariate association with linear models (MaAsLin2)^54^. Calculated coefficients, *P-*value, and adjusted *P*-value (*q*-value) were used for analysis.

#### Random forest analysis

The random forest model was used to identify genus-level features differing between HCs and patients with MS using the R package, “randomForest”^55^. Genus-level relative abundance tables from the Chinese and Italian cohorts were first merged into a single feature table, and cohort identity was retained as sample metadata to facilitate downstream interpretation and sensitivity checks but was not included as a predictive feature. To reduce the influence of cohort-specific technical effects, samples were randomly split into training (70%) and test (30%) sets stratified by the disease group. Model tuning was performed on the training set using the “*tuneRF*” function to select the optimal number of variables considered at each split (mtry), whereas the number of trees (ntree) was set to a sufficiently large value to ensure stable performance estimates. The final classifier was trained on the training set and evaluated on the held-out test set. Feature prioritization was performed using tenfold cross-validation with the “*rfcv*” function, ranking genera by variable importance. Discriminatory performance was assessed by receiver operating characteristic analysis using the “*plot.roc*” function in the “pROC” package^56^, and 95% confidence intervals were computed with the “*ci.se*” function using 2,000 stratified bootstrap replicates.

#### Inter-variation and intra-variation analysis

Inter-variation and intra-variation in the microbiomes were analyzed based on Bray–Curtis dissimilarity calculated using the “*vegdist*” function R package “vegan” (v.2.6-4)^57^. This analysis was used to determine which part of the GI tract had relatively more perturbated microbiota upon EAE induction.

#### Network analysis

Interspecies correlations were estimated using Spearman correlation analysis. The corresponding correlation network was visualized using the R package “qgraph”^58^. To identify network hub taxa which can modulate the microbiota of the cecum and colon, network analysis was performed on the samples of *V. ratti* MHL0042-treated EAE-induced mice. Node sizes were scaled based on the PageRank algorithm centrality measure, which was determined using the R package “igraph”^59^. Edge thickness denoted using a Spearman correlation *rho* and only significant correlations (with a *rho* > |0.7| and *P* < 0.05) were shown in the network. The top 10 species with the highest PageRank were selected as hubs in the networks.

#### Multiscale potential of heat diffusion for affinity-based transition embedding analysis

To study the differentiation and transmission of each GI tract microbiota, we determined multiscale potential of heat diffusion for affinity-based transition embedding (MS-PHATE) using the python-package “multiscale_phate” (v.0.0)^60^. The MS-PHATE run on GI tract samples was performed using the following parameters: *random_state* = 2L, *n_pca* = 50L, *decay* = 15L, and *knn* = 20L. Manifold density estimation was used in the Python package “meld” (v.1.0.0)^61^, to identify “hotspots”, which represents each cluster associated with the GI tract prevalence as well as network hubs. The sample-associated relative likelihood approach was used to determine the densities of GI tract samples and network hubs for each sample within MS-PHATE.

### Isolation of lamina propria and CNS lymphocytes

The small intestine and colon tissues were dissected, and fat and luminal contents were removed by flushing with ice-cold PBS (Corning, NY, USA). The tissues were minced and sequentially incubated in Hank’s Balanced Salt Solution (Gibco, Waltham, MA, USA) containing 2% fetal bovine serum (FBS; Corning), 10 mM HEPES (Gibco), 5 mM ethylenediaminetetraacetic acid (Sigma-Aldrich), and 1 mM dithiothreitol (Thermo Fisher Scientific). This was followed by enzymatic digestion in Roswell Park Memorial Institute (RPMI) 1640 medium (Corning) containing 1 mg/ml collagenase (Sigma-Aldrich), 20 µg/ml DNase I (Sigma-Aldrich), and 1.25 mg/ml dispase (Roche, Basel, Switzerland). The dissociated tissue suspensions were subjected to a Percoll (GE Healthcare, Chicago, IL, USA) density gradient, and lamina propria lymphocytes (LPLs) were collected from the interface layer.

For CNS lymphocyte isolation, brain and spinal cord tissues were dissected and minced using the same protocol as that used for LPL isolation. Isolated cells were counted and stored in ice-cold complete medium consisting of RPMI 1640 supplemented with 10% heat-inactivated FBS, 1% non-essential amino acids (Gibco), 1% sodium pyruvate (Gibco), 1% penicillin/streptomycin (Corning), and 0.1% 2-mercaptoethanol (Thermo Fisher Scientific).

### CNS cell isolation for microglial analysis

Brain and spinal cord tissues were dissected and minced; microglia were isolated following a previously published protocol^62^. Briefly, the tissues were dissociated in homogenization medium, RPMI-1640 supplemented with 2% heat-inactivated FBS, and passed through a 70-μm cell strainer. The resulting cell suspension was centrifuged at 400×*g* for 6 min at 4 °C, and the pellet was resuspended in 40% Percoll prepared in homogenization medium. This suspension was then layered over 80% Percoll in the same medium. After centrifugation at 1000×g for 20 min at 18 °C, the interphase layer containing microglia was collected. The isolated cells were washed with homogenization medium, and the resulting cell pellet was used for further analysis.

### Flow cytometry

Cells isolated from LPLs, lymph nodes, and CNS were incubated with 50 ng/ml phorbol 12-myristate 13-acetate (Sigma-Aldrich) and 750 ng/ml calcium ionophore (Sigma-Aldrich) in the presence of GolgiPlug (BD Biosciences, San Jose, CA, USA) for 4 h. For MOG-specific cell analysis, cells were re-stimulated with 50 µg/ml MOG_35–55_ peptide for 24 h before flow cytometry. The live/dead cells were gated using LIVE/DEAD^TM^ fixable violet dead cell stain kit (Thermo Fisher Scientific). Cells were stained with the following fluorochrome-conjugated antibodies. For microglia, surface and intracellular staining was performed using CD45-Brilliant Violet 510, CX3CR1-APC/Cyanine7 (BioLegend, San Diego, CA, USA), CD11b-APC, and CD68-PE (all from eBioscience, CA, USA; unless otherwise specified). CD4⁺ T cells were stained using CD4-PerCP-Cy5.5, IL-17A-APC, IFN-γ-FITC, and FOXP3-PECy7 (eBioscience). Samples were evaluated using a MACSQuant® Analyzer 10 flow cytometer (Miltenyi Biotec, Auburn, CA, USA), and data were analyzed using the FlowJo software (Tree Star Inc., Ashland, OR, USA).

### Immunofluorescence staining

Mice were perfused with 4% paraformaldehyde (Geneall Biotechnology, Seoul, Korea), and spinal cords were carefully dissected. The collected tissues were fixed overnight in 4% paraformaldehyde at 4 °C, followed by cryoprotection in 30% sucrose solution for 24 h at 4 °C. For frozen sectioning, tissues were embedded in optimal cutting temperature compound (Sakura Finetek USA, Inc., Torrance, CA, USA) and sectioned at a thickness of 10 μm. The sections were permeabilized with 0.5% Triton X-100 in PBS for 10 min and then blocked with 1% bovine serum albumin in PBS for 1 h at room temperature. The samples were incubated overnight at 4 °C with the following primary antibodies: anti-Iba1 monoclonal antibody (GT10312, Invitrogen, Carlsbad, CA, USA) and anti-CD68 monoclonal antibody (FA-11, Invitrogen). After washing three times with ice-cold PBS, the sections were incubated overnight at 4 °C with Alexa Fluor 488-conjugated anti-mouse and Alexa Fluor 555-conjugated anti-rat secondary antibodies (Invitrogen). The nuclei were counterstained with 4′,6-diamidino-2-phenylindole (DAPI) (Sigma-Aldrich). Images were acquired using a fluorescence microscope (Leica DMi8, Leica Microsystem, Wetzlar, Germany) at the Soonchunhyang Biomedical Research Core Facility of the Korea Basic Science Institute. Iba1-positive and CD68-positive cells were quantified using the ImageJ software and expressed as both the number and percentage of positive cells.

### Collection of cecal content and global metabolome profiling

The cecal contents were aseptically collected at the peak of disease for global metabolome profiling. Metabolites were extracted from mouse cecum samples using liquid–liquid extraction^63^. Briefly, 400 µl of chloroform/methanol (2/1, v/v) was added to the tissues and homogenized using TissueLyzer (Qiagen), followed by centrifugation for 15 min. In the absence of phase separation, additional 100 µl of chloroform or water was added. Lipid-containing nonpolar metabolites were collected from the lower organic phase, and polar metabolites were collected from the upper aqueous phase. The organic or aqueous solution was then dried using a vacuum centrifuge and stored at −20 °C, until further analysis. The dried matter was reconstituted with 50 μl of mobile phase A (0.1% formic acid in H_2_O) before liquid chromatography (LC)–mass spectrometry.

An LC–mass spectrometry system equipped with Dionex Ultimate3000 and LTQ-Orbitrap XL (Thermo Fisher Scientific) was used for metabolome profiling in the positive and negative ion mode. A reverse-phase column (Pursuit5 C18, 3 μm, 150 × 2.1 mm) was used to analyze the organic phase solutions using mobile phase A (0.1% formic acid in H_2_O) and mobile phase B (0.1% formic acid in methanol). LC was performed at 200 µl/min and 25 °C. The separation gradient was as follows: 75% to 99.9% B for 2 min, hold at 99.9% B for 13 min, 99.9% to 75% B for 0.5 min, and finally hold at 75% B for 4.5 min. A hydrophilic interaction LC column (Waters XBridge BEH amide, 2.5 μM, 2.1 × 150 mm) was used to analyze the aqueous phase solutions using mobile phases A (10 mM ammonium acetate, 10 mM ammonium hydroxide in H_2_O/acetonitrile (v/v, 95/5), pH 9) and B (10 mM ammonium acetate, 10 mM ammonium hydroxide in H_2_O/acetonitrile (v/v, 5/95), pH 7). LC was performed at 200 µl/min and 25 °C. The separation gradient was as follows: hold at 70% B for 1 min, 70% to 40% B for 5 min, hold at 40% B for 6 min, 40% to 70% B for 0.1 min, and finally hold at 70% B for 7.9 min.

Data normalization was performed using log transformation and auto-scaling. Metabolite features, including neutral mass and retention time values, were determined using Compound Discoverer 3.2. Significantly altered metabolite features (fold change > 1.2 or < 0.8, with *P* < 0.05) were selected and identified using the METLIN and KEGG database, with a mass accuracy of 10 ppm.

A volcano plot was used to visualize significantly different metabolites of the cecum among the PBS, EAE, and EAE + *V. ratti* MHL0042 groups. The *x*-axis presents the MaAsLin2 coefficients, and the *y*-axis presents significance with −log10(*P*-value), which resulted from MaAsLin2. The threshold was set at coefficients > |0.05| and *q*-value > 0.05 in the metabolite analysis. The peak area of each metabolite obtained from the non-targeted metabolite analysis was normalized using the variance stabilizing normalization method^64^.

### Collection of cecal content, serum, and spinal cord and LC–mass spectrometry

Cecal content was collected aseptically and lyophilized overnight. Serum was collected through cardiac puncture and separated from the blood cells using 10,000×*g* for 10 min at 4 °C. Cervical spinal cord was isolated from the mice. All samples were stored in −80 °C until further analysis.

To validate the enrichment of DOPE identified during non-targeted metabolomics analysis, targeted quantification was conducted using an LC–mass spectrometry system (Shimadzu, Kyoto, Japan) equipped with a binary pump (LC-20AD), autosampler (SIL-20A), degasser (DGU-20A), and mass spectrometer (MS-2020). Chromatographic separation was performed by injecting 1 μl of each sample into a C18 reverse-phase column (Vydac, 5 μm, 2.1 × 150 mm; Hesperia, CA, USA) maintained at 60 °C, with a flow rate of 0.2 ml/min. Mobile phase A consisted of water/methanol/acetonitrile (1:1:1, v/v/v), and mobile phase B consisted of isopropanol/acetonitrile (5:1, v/v), both containing 5 mM ammonium acetate. The gradient elution program was set as follows: 0–0.5 min, 20% B; 0.5–1.5 min, 40% B; 1.5–3 min, 60% B; 3–13 min, 98% B; 13–13.1 min, 20% B; and 13.1–17 min, 20% B. Mass spectrometric detection was performed using an electrospray ionization source operated in negative-ion selected ion monitoring mode targeting DOPE at *m/z* 742.5. The desolvation line temperature was set to 250 °C, and heat block temperature was maintained at 200 °C. The nebulizing and drying gas flow rate were 1.5 l/min and 15 l/min, respectively, using high-purity nitrogen. DOPE levels were quantified by integrating peak areas from negative-ion selected ion monitoring mode chromatograms and converting them into concentration values using a calibration curve generated from standards.

### Cecal content phospholipase activity colorimetric analysis

Phospholipase activity was determined using Phospholipase A2 activity assay kit (Abcam, Cambridge, UK), according to the manufacturer's protocol. Briefly, cecal contents were taken aseptically and diluted to 1 mg/μl in PLA2 assay buffer. Cecal contents were spun down to separate the supernatant from the pellet. Each well was filled with 10 μl reconstituted-5,5’-dithio-bis-(2-nitrobenzoic acid), 10 μl cecal content supernatants, and 5 μl assay buffer. The reaction was initiated by adding 200 μl reconstituted substrate. The absorbance was read at 414 nm every minute after the reaction began.

### In vitro co-culture of immune cells with *V. ratti* MHL0042

Immune cells were isolated from the mesenteric lymph nodes and spleen of EAE-induced mice 13 days post-immunization. The cells were subsequently cultured ex vivo in standard bacterial culture medium supplemented with 150 μg/ml gentamicin in the presence or absence of *V. ratti* MHL0042 for 24 h. Each culture condition contained 2.5 × 10^6^ cells and, where applicable, 2.5 × 10^8^ CFU of *V. ratti* MHL0042, representing a bacteria-to-cell ratio of 100:1. Following incubation, the cells were harvested for RNA isolation, and expression levels of *Il10* and *Il12p40* were quantified using reverse transcription (RT)-PCR. Gene expression in cells cultured with *V. ratti* MHL0042 was normalized to that of cells cultured in its absence, with the latter designated as the reference value of 1; the results were expressed as fold change relative to this reference.

### RT-PCR analysis of host tissues and cells

For cell samples, the total RNA was extracted directly using TRIzol reagent (Invitrogen). The indicated tissue samples were homogenized using the Mini-BeadBeater-24 (Biospec Products, Bartlesville, OK, USA), followed by RNA extraction with TRIzol reagent. All RNA samples were treated with DNase I (Sigma-Aldrich) to eliminate genomic DNA contamination. Complementary DNA was synthesized using the ReverTra Ace® qPCR RT Master Mix (TOYOBO Co., Ltd., Osaka, Japan), according to the manufacturer’s instructions. RT-PCR was performed using the SYBR Green Real-Time PCR Master Mix Kit (TOYOBO) with primers listed in Supplementary Table 1. Reactions were performed on a QuantStudio™ real-time PCR system (Applied Biosystems, Foster City, CA, USA). Gene expression levels were normalized to those of β-actin and reported as relative expression values.

### Demyelination analysis using Luxol fast blue staining

Ten micrometers of paraffin-embedded cervical spinal cord sections were used for demyelination analysis using Luxol fast blue (LFB) staining. Tissue sections were deparaffinized and hydrated through a series of 95% ethanol solutions. The sections were then incubated overnight in LFB solution (Sigma-Aldrich) at 60 °C. Subsequently, the sections were thoroughly rinsed in 95% ethanol followed by distilled water. For differentiation, the sections were repeatedly dipped in 0.05% lithium carbonate solution (Sigma-Aldrich) and sequentially rinsed in 70% ethanol until the gray matter became colorless and white matter remained blue. The sections were then rinsed in distilled water and counterstained with 0.1% Cresyl Violet solution (Abcam) for 1 min. After counterstaining, the sections were immediately rinsed in distilled water, dehydrated through an ethanol–xylene gradient, and mounted using Permount™ mounting medium (Fisher Scientific, Loughborough, UK). The slides were analyzed microscopically (Nikon Instruments Inc., Melville, NY, USA), and the optical density of LFB was determined using the ImageJ software, to calculate the percentage of white matter demyelination.

### Treatment of microglia with DOPE

The immortalized microglial (IMG; Sigma-Aldrich) cell line was cultured in high glucose Dulbecco’s modified Eagle’s medium (Sigma-Aldrich) supplemented with 10% FBS, 1% penicillin/streptomycin, and 1% L-glutamine (Gibco). The IMG cells were treated with varying concentrations of DOPE in the presence or absence of 5 ng/ml IFN-γ (PeproTech Inc., Rocky Hill, NJ, USA) for 24 h. Subsequently, the supernatants and cell pellets were collected for cytokine quantification and gene expression analysis, respectively.

### Cell immunofluorescence staining

Following DOPE treatment, the IMG cells were sequentially washed with PBS, fixed with 4% PFA, and blocked with 5% BSA in PBS for 1 h at room temperature. The samples were then incubated overnight at 4 °C with anti-CD11b (ab184308, Abcam) and anti-CD68 (FA-11, Invitrogen) monoclonal antibodies. Following three washes with PBS, the cells were incubated with Alexa Fluor 488-conjugated anti-rabbit and Alexa Fluor 555-conjugated anti-rat secondary antibodies (both from Invitrogen) for 1 h at room temperature. The nuclei were counterstained with DAPI (Sigma-Aldrich). Images were acquired using a fluorescence microscope (Leica DMi8) at the Soonchunhyang Biomedical Research Core Facility of the Korea Basic Science Institute. The CD11b-positive and CD68-positive areas were quantified using the ImageJ software and expressed as fluorescence intensity.

### Determination of cytokine concentration using the enzyme-linked immunosorbent assay

The supernatants from ex vivo cultures of MOG-specific T cells or microglial cell line cultures were collected for cytokine quantification. Total concentrations of IL-17A and IFN-γ were measured using the mouse IL-17A and IFN-γ uncoated enzyme-linked immunosorbent assay kits, respectively (both from eBioscience), according to the manufacturer’s instructions.

### RNA sequencing

Two biological replicates of IMG cells were treated with vehicle or 25 μM DOPE in the presence of 5 ng/ml IFN-γ for 24 h. Total RNA was isolated using TRIzol reagent and treated with DNase I. DNBseq-stranded mRNA library was constructed by BGI Genomics Co., Ltd. RNA was sequenced on the DNBseq™ platform. The low quality, adaptor contamination, and high levels of unknown base N reads were filtered before bioinformatics analysis. The clean reads were mapped onto the mouse reference genome (*Mus musculus*, NCBI_GCF_000001635.27_GRCm39) using Hierarchical Indexing for Spliced Alignment of Transcript. For differentially expressed gene (DEG) analysis, clean reads were aligned to the reference genes using Bowtie2 and quantified using RSEM tools. DEGs were detected using the average of normalized counts divided by size factors using DEseq2 algorithm (log2FoldChange > 1.5 and *q*-value < 0.05).

### Statistical analyses

Phenotypical observations were statistically analyzed using the GraphPad software (PRISM 10 GraphPad, San Diego, CA, USA) with Student’s *t* test and one-way analysis of variance. A *P*-value of ≤ 0.05 was considered statistically significant. All data presented in each experiment are representative results from three independent biological experiments.

For microbiome statistical analysis, pairwise comparison in the two different groups was performed using Wilcoxon-rank sum test. Differences in β-diversity were visualized through non-metric multidimensional scale plots based on Bray–Curtis dissimilarity. Significance was determined using PERMANOVA (Adonis2 from the package vegan with 999 permutations). The correlation analysis between satellite microbiota and k-gene was performed using the Spearman correlation with “*rcorr*” function in the “Hmisc” R package^65^. The correlogram was visualized using the “corrplot” R package^66^.

## Results

### *V. ratti* MHL0042 reduces EAE severity and demyelination

Previous studies have reported that *Veillonella* (Veillonellaceae family) is less abundant in patients with MS than in HCs, and *V. ratti* can modulate the gut microbiome and influence host immune responses^20,25^. To assess the clinical relevance of targeting *V. ratti* in MS, we analyzed three publicly available MS gut microbiome cohorts (iMSMS^28^, Italian^26^, and Chinese cohorts^27^), processing 16S amplicon and WMS datasets separately. Alpha diversity patterns differed across cohorts, potentially reflecting ethnicity, sample size, and technical variation (Supplementary Fig. 1a). By contrast, β-diversity differed consistently between patients with MS and HCs in all cohorts (*P* < 0.001; Supplementary Fig. 1b). In 16S datasets, both random forest classification and MaAsLin2 differential abundance analysis identified depleted *Veillonella* abundance in patients with MS (Supplementary Fig. 1c,d), and this reduction was also observed in the WMS datasets (Supplementary Fig. 1e). Together, these analyses support the translational relevance of using *V. ratti* as a mechanistically tractable candidate to probe microbiome–immune interactions in an EAE model.

On the basis of these human-cohort observations, we next examined the immunomodulatory potential of *V. ratti* MHL0042, a strain isolated from human infant feces. Total immune cells from the mesenteric lymph nodes and spleen were cultured in the presence of *V. ratti* MHL0042, and mRNA expression levels of the anti-inflammatory cytokine, *Il10*, and pro-inflammatory cytokine, *Il12p40*, were quantified relative to those of untreated controls. *V. ratti* MHL0042 exposure preferentially increased *Il10* expression compared with that of *Il12p40* (Supplementary Fig. 2), indicating anti-inflammatory effects induced by modulating cytokine expression.

Given the consistent depletion of *Veillonella* abundance in patients with MS and the anti-inflammatory effects induced by *V. ratti* MHL0042 ex vivo, we hypothesized that this strain could modulate the development and severity of EAE. To test this, mice were orally administered *V. ratti* MHL0042 daily for 1 week before EAE induction and the dose continued throughout the experiment until the peak of disease. EAE-induced mice receiving PBS served as controls. Clinical symptoms were assessed daily using a standard EAE scoring system ranging from 0 (no signs) to 5 (complete paralysis).

**Fig. 1:**
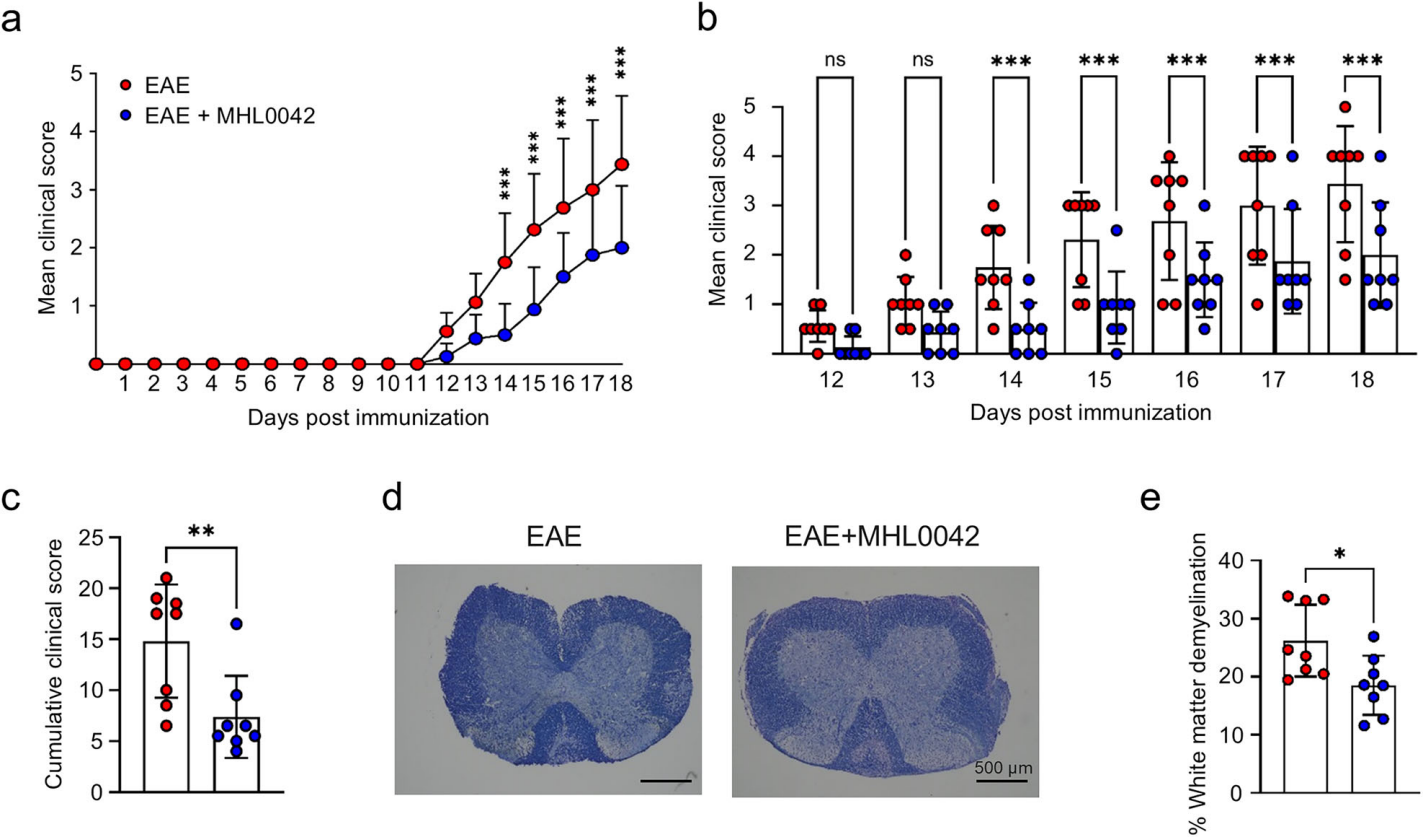
Administration of *Veillonella ratti* MHL0042 protects mice from developing severe experimental autoimmune encephalomyelitis. **a**,**b** Mean clinical scores of experimental autoimmune encephalomyelitis (EAE) mice treated with or without *V. ratti* MHL0042 from the day of EAE induction to 18 days post-immunization. **c** Cumulative clinical scores of EAE mice with or without *V. ratti* MHL0042 treatment. **d** Representative Luxol fast blue staining of myelin in spinal cord sections. **e** Quantification for the percentage of white matter demyelination in the spinal cord. Results are expressed as mean ± standard deviation from EAE (*n* = 8) and EAE + *V. ratti* MHL0042 (*n* = 8) groups, *P < 0.05; **P < 0.01; ***P < 0.001. ns, not significant.

Treatment with *V. ratti* MHL0042 significantly suppressed disease severity in EAE mice. *V. ratti* MHL0042-treated mice exhibited significantly lower mean clinical scores than those of PBS-treated controls, beginning on day 12 post-immunization and persisting through the disease peak at day 18 (*P* < 0.001, Fig. 1a,b). In addition, the cumulative clinical scores were significantly reduced in *V. ratti* MHL0042-treated EAE mice compared with those in the controls (*P* < 0.01, Fig. 1c).

To evaluate demyelination, a hallmark of CNS pathology in MS^67^, spinal cords were collected and stained with LFB. The extent of demyelination was quantified by measuring the white matter area. PBS-treated EAE mice exhibited widespread demyelination across the dorsal, lateral, and ventral funiculi. By contrast, *V. ratti* MHL0042-treated mice showed demyelination predominantly restricted to the dorsal funiculus (Fig. 1d). Consistent with the clinical scores, the percentage of demyelinated CNS area was significantly lower in *V. ratti* MHL0042-treated mice compared with that in untreated EAE controls (*P* < 0.05, Fig. 1e). Thus, oral *V. ratti* MHL0042 administration confers protection against EAE pathogenesis, attenuating both clinical symptoms and CNS demyelination.

### *V. ratti* MHL0042 modulates gut microbiota during EAE development

To investigate whether *V. ratti* MHL0042 modulates the gut microbiota to confer resistance against EAE, we performed region-specific microbiota analysis along the GI tract, including the proximal small intestine, distal small intestine, cecum, colon, and feces, during EAE progression. This approach was designed to localize the anatomical sites where *V. ratti* MHL0042 induces the most prominent community changes and to prioritize the region for downstream analyses integrating microbiome remodeling with metabolite alterations and functional potential.

Although overall bacterial diversity and total bacterial amounts changed similarly across all groups, interorgan differences in the microbiota were reduced in both EAE-induced groups (EAE and EAE + *V. ratti* MHL0042) (Supplementary Fig. 3a,b). Non-metric multidimensional scale plots based on Bray–Curtis distances revealed significant microbiota variation among organs across all groups (*P* < 0.001; Supplementary Fig. 3c); however, organ separation was attenuated in the EAE (*R*^2^ = 0.53) and EAE + *V. ratti* MHL0042 (*R*^2^ = 0.52) groups compared with those in the controls (*R*^2^ = 0.89). Notably, the relative abundance of Bacteroidota was reduced in the cecum, colon, and feces of EAE-induced mice relative to that in the controls (Supplementary Fig. 3d).

**Fig. 2:**
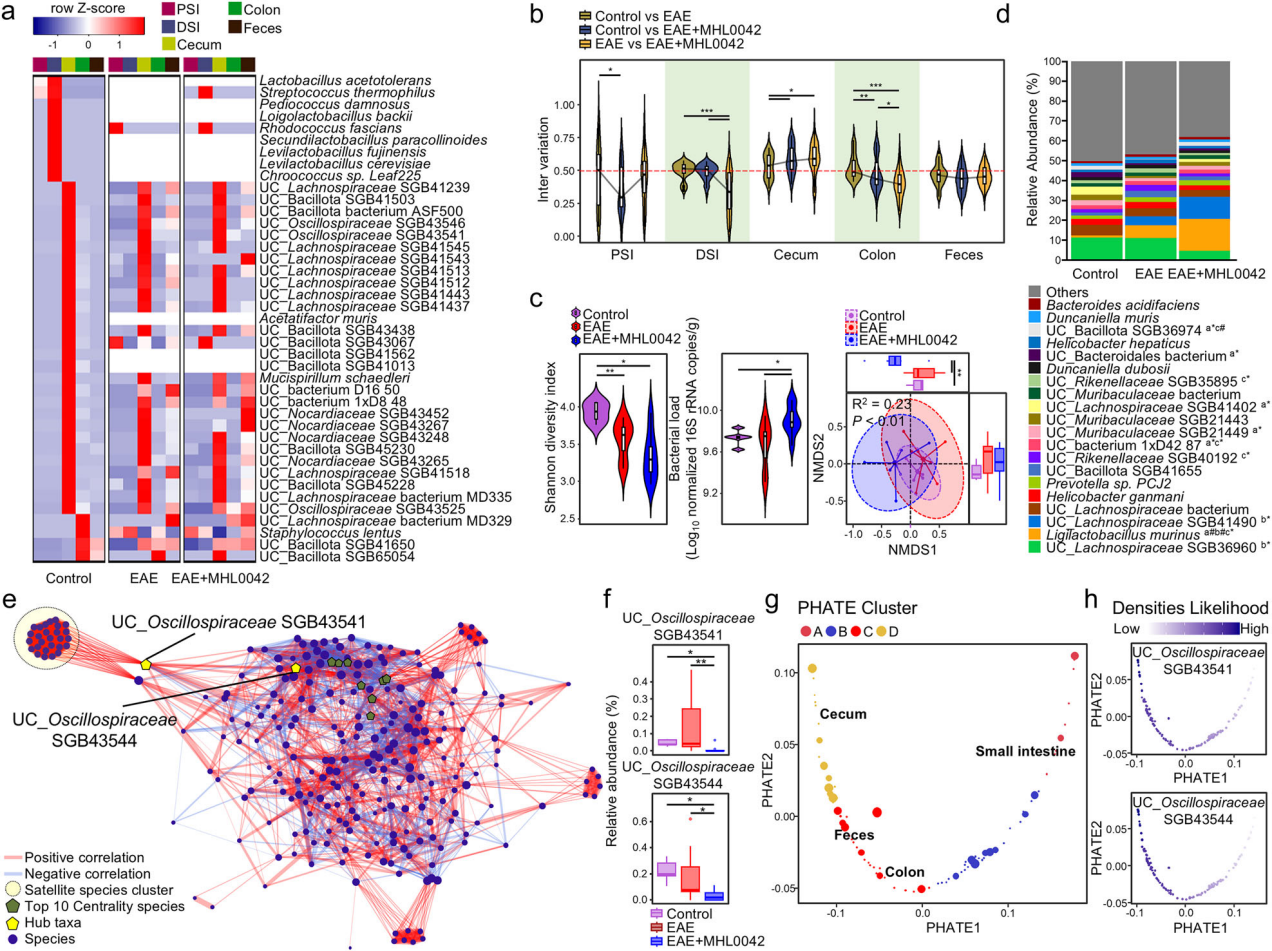
Gut microbiota comparisons across gastrointestinal tract regions in the three groups. **a** Indicator species defined in control mice were compared across groups based on the gastrointestinal (GI) region. **b** Bray–Curtis dissimilarity was used to assess intergroup differences in each GI region. Significance was tested using Dunn′s test with false discovery rate correction. **c** Cecal microbial diversity and total bacterial load were compared among the groups. Non-metric multidimensional scale (NMDS) based on Bray–Curtis dissimilarity visualizes species-level composition. PERMANOVA and Dunn's test (false discovery rate correction) were used for statistical comparisons. **d** Relative abundance of dominant species in the cecal microbiota. Bars show group means; species <1% mean abundance were grouped as “Others”. MaAsLiN2 was used for significance testing. Comparisons: control versus EAE (part **a**); control versus EAE + *Veillonella ratti* MHL0042 (part **b**); EAE versus EAE + *V. ratti* MHL0042 (part **c**). *P < 0.05; ^#^*q* < 0.05. **e** Network of cecal microbiota in the EAE + *V. ratti* MHL0042 group. Edges: significant Spearman correlations (|*ρ*|> 0.7, *P* < 0.05); red: positive, blue: negative; thickness: correlation strength. Node size: PageRank centrality. Green pentagons: top 10 central species; yellow: downregulated network hub taxa; dashed circles: associated satellite. **f** Relative abundance of selected network hub taxa across treatment groups. Statistical differences in relative abundance were assessed using MaAsLin2 to evaluate group-specific enrichment. *P < 0.05; **P < 0.005. **g** Multiscale potential of heat diffusion for affinity-based transition embedding (PHATE) showing microbiota clustering across GI regions. Labels A–D indicate that four clusters denote PHATE diffusion–condensation cluster. **h** Manifold density estimation (MELD) likelihoods visualization of network hub taxa density on PHATE embedding. DSI, distal small intestine; EAE, experimental autoimmune encephalomyelitis; PSI, proximal small intestine.

We next assessed organ-specific microbial perturbations using indicator species analysis (Fig. 2a). In the controls, each gut region harbored distinct indicator species, whereas these region-specific patterns were disrupted in both the EAE and EAE + *V. ratti* MHL0042 groups. Intergroup differences were further evaluated within each site using Bray–Curtis distances (Fig. 2b). Although microbiota perturbations were evident from the proximal small intestine to feces, the cecum showed the most pronounced separation between the EAE and EAE + *V. ratti* MHL0042 groups, indicating that this compartment is a major site of *V. ratti* MHL0042-associated microbiota remodeling. Accordingly, we focussed subsequent analyses on the cecum.

In the cecum, the EAE + *V. ratti* MHL0042 group exhibited significantly reduced microbial diversity but increased bacterial load relative to those in the other groups (*P* < 0.05; Fig. 2c). Cecal microbiota composition differed significantly among groups (*R*^2^ = 0.23, *P* < 0.01). In the EAE + *V. ratti* MHL0042 group, the relative abundance of uncultured (UC)_Lachnospiraceae SGB36960 decreased, whereas that of *Ligilactobacillus murinus* and UC_Lachonospiraceae SGB41490 increased (*P* < 0.05; Fig. 2d). Collectively, these findings indicate that *V. ratti* MHL0042 administration is associated with distinct cecal microbiota remodeling, including reduced microbial diversity and selective shifts in specific bacterial taxa in the EAE model.

As microbiota differences can reflect altered microbial interactions, we next analyzed whether interspecies relationships are reshaped in the cecum following *V. ratti* MHL0042 administration. Network analysis of the cecal microbiota (Fig. 2e) identified the top 10 central taxa using the PageRank algorithm (Supplementary Table 2). Among these, UC_Oscillospiraceae SGB43541 and SGB43544 were network hub taxa within a satellite cluster (SC) and showed strong positive correlations with other species in the SC. The relative abundances of these two hub taxa were significantly reduced in the EAE + *V. ratti* MHL0042 group compared with those in the EAE group (*P* < 0.05; Fig. 2f). Thus, *V. ratti* MHL0042 administration co-occurred with reduced SC connectivity and negative associations with hub taxa, consistent with the attenuation of SC interactions.

**Fig. 3:**
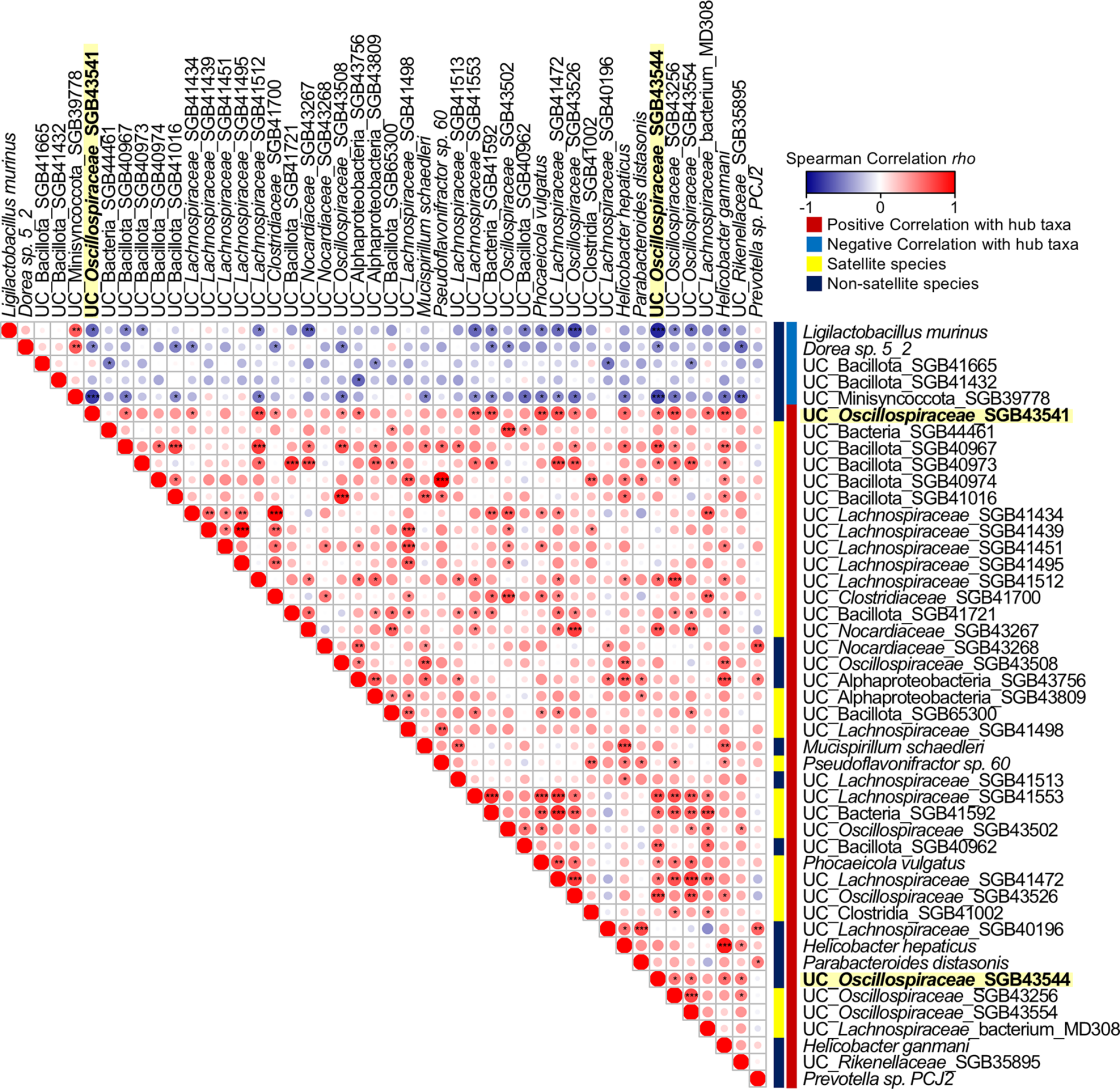
Association between network hub taxa and co-modulated microbial communities in *Veillonella ratti* MHL0042-treated experimental autoimmune encephalomyelitis mice cecum. Correlogram illustrating modulated species associated with the network hub taxa using Spearman correlation analysis. The color intensity and square size reflect the strength of the coefficients. *P < 0.05; **P < 0.005; ***P < 0.0005.

To examine whether the cecum-centered signatures extend to distal compartments, we performed MS-PHATE and manifold density estimation analyses. The cecal microbiota clustered closely with those of the colon and feces, indicating possible downstream similarity or microbial migration (Fig. 2g). Consistently, PHATE analysis revealed that the network hub taxa (UC_Oscillospiraceae SGB43541 and SGB43544) showed similar spatial clustering patterns across the cecum and distal sites (Fig. 2h). Network analysis of the colon microbiota also identified hub taxa using the PageRank algorithm (Supplementary Fig. 4a and Supplementary Table 2), and three hub taxa, including UC_Oscillospiraceae SGB43544, differed significantly among groups. Notably, SGB43544, which acted as a hub in the cecum, also correlated positively with an SC in the colon (Supplementary Fig. 4a,b), and taxa within this SC were reduced following *V. ratti* MHL0042 administration (Fig. 3).

Collectively, region-resolved profiling identified the cecum as a primary site of *V. ratti* MHL0042-associated restructuring, with coordinated shifts in network hub taxa (SGB43544), which also reflected in the distal compartments.

### *V. ratti* MHL0042 administration suppresses neuroinflammation in EAE

Considering the observed microbiota alterations in *V. ratti* MHL0042-treated EAE mice, we sought to elucidate whether these changes influence EAE pathogenesis by modulating inflammation. In small intestinal and colonic tissues, pro-inflammatory cytokine genes (*Il17a*, *Il1b*, *Il6*, *Tnfa*, and *Ifng*) were not significantly upregulated, and *V. ratti* MHL0042 administration did not significantly alter these cytokines (data not shown).

To assess whether *V. ratti* MHL0042 influences the inflammatory properties of CD4^+^ T cells, we analyzed specific subsets of CD4^+^ T cells (CD4^+^IL-17A^+^, CD4^+^IFN-γ^+^, and CD4^+^FOXP3^+^) in the small intestinal lamina propria and spinal cord. Lymphocytes were isolated from both sites and stimulated in vitro with phorbol 12-myristate 13-acetate and ionomycin. The proportions of these T cell subsets did not differ significantly between the EAE and EAE + *V. ratti* MHL0042 groups in either small intestinal LPLs (Supplementary Fig. 5a,b) or the spinal cord (Supplementary Fig. 5c,d), suggesting that the *V. ratti* MHL0042-induced attenuation of EAE symptoms may not be due to the direct inhibition of pro-inflammatory CD4^+^ T cell populations.

**Fig. 4:**
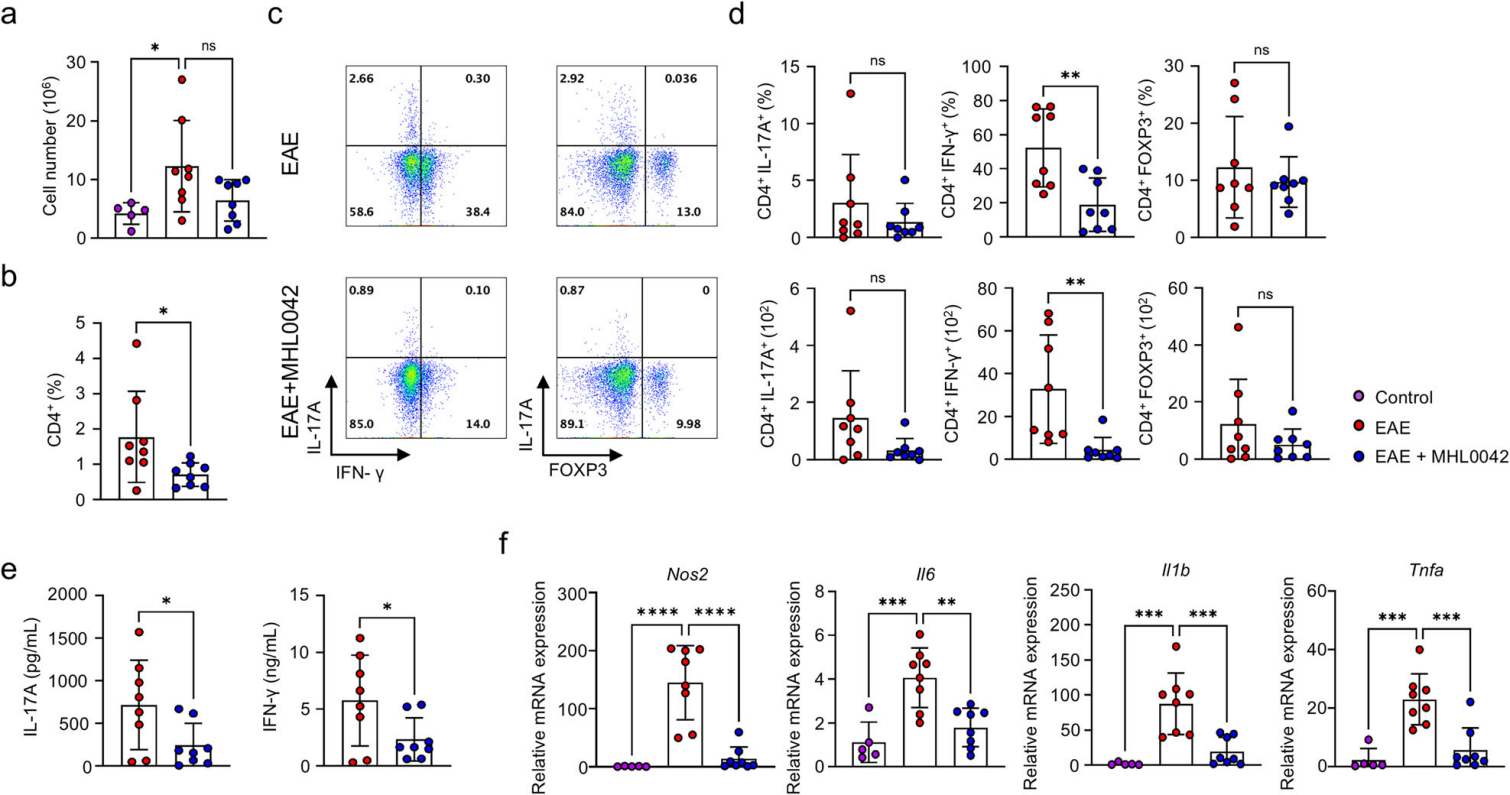
Treatment with *Veillonellaratti* MHL0042 modulates the central nervous system immune environment. Spinal cord-infiltrating lymphocytes were isolated and re-stimulated with the MOG_35–55_ peptide. **a** Total number of central nervous system-infiltrating cells isolated from control, experimental autoimmune encephalomyelitis (EAE), and *V. ratti* MHL0042-treated EAE mice. **b** The frequency of MOG-specific CD4⁺ T cells isolated from EAE mice and *V. ratti* MHL0042-treated EAE mice calculated as a percentage of total live cell population. Specific CD4^+^ cell lineages, CD4⁺IL-17A⁺, CD4⁺IFN-γ⁺, and CD4⁺FOXP3⁺ cells, were then analyzed using flow cytometry under live CD4⁺ gating. **c** Representative fluorescence-activated cell sorting plot analysis. **d** The frequencies and numbers of specific lineages of CD4^+^ cells, CD4⁺IL-17A⁺, CD4⁺IFN-γ⁺, and CD4⁺FOXP3⁺ cells, calculated as percentages of CD4⁺ T cells. **e** IL-17A and IFN-γ concentrations in culture supernatants. Culture supernatants from MOG_35–55_-re-stimulated spinal cord-infiltrating lymphocytes were collected for enzyme-linked immunosorbent assay-based quantification of secretory pro-inflammatory cytokines. **f** Assessment of central nervous system inflammatory responses was conducted using reverse transcription-PCR. Relative mRNA expression levels of *Nos2*, *Il6*, *Il1b*, and *Tnfa* in spinal tissues from control, EAE, and *V. ratti* MHL0042-treated EAE mice. Data are presented as mean ± standard deviation from control (*n* = 5), EAE (*n* = 8), and *V. ratti* MHL0042-treated EAE (*n* = 8) groups, *P < 0.05; **P < 0.01; ***P < 0.001; ****P < 0.0001. IFN, interferon; MOG, myelin oligodendrocyte glycoprotein; ns, not significant; TNF, tumor necrosis factor.

As increased inflammatory immune cell infiltration in the CNS is a hallmark of EAE, and antigen-specific CD4^+^ T_H_1 and T_H_17 cells are major drivers of neuroinflammation in MS^68,69^, we hypothesized that *V. ratti* MHL0042 may act locally within the CNS to suppress antigen-specific pathogenic T_H_1 and T_H_17 cells. To test this hypothesis, spinal cord-derived lymphocytes were re-stimulated with the MOG peptide ex vivo, and CD4^+^ T cell subsets were assessed. The total number of CNS-infiltrating cells and percentage of MOG-specific CD4^+^ T cells showed a modest reduction in the treatment group (Fig. 4a,b). The frequency and the total number of MOG-specific CD4^+^IFN-γ^+^ cells in the CNS were also significantly reduced in the *V. ratti* MHL0042-treated group compared with those in the untreated EAE group (Fig. 4c,d). Moreover, production of the pro-inflammatory cytokines, IL-17A and IFN-γ, was significantly lower in cultures derived from *V. ratti* MHL0042-treated EAE mice (Fig. 4e), suggesting that *V. ratti* MHL0042 attenuates EAE severity by reducing CNS-resident antigen-specific pathogenic CD4^+^ T cells.

Finally, we assessed CNS inflammation by quantifying pro-inflammatory cytokine gene expression in spinal cord tissue. EAE induction significantly upregulated *Nos2*, *Il6*, *Il1b*, and *Tnfa* expressions, whereas *V. ratti* MHL0042 administration significantly reduced the expression levels of these cytokines to those similar to the control (Fig. 4f). Thus, *V. ratti* MHL0042 mitigates neuroinflammation by suppressing antigen-specific effector T cell expression and inflammatory cytokine production in the CNS.

### *V. ratti* MHL0042 administration suppresses microglial activation in EAE

In addition to the central role of pathogenic CD4^+^ T cells in EAE pathogenesis, microglia also contribute significantly to demyelination in both MS and EAE models^70^. Notably, microglial depletion delays EAE onset^71^, and microglia-driven neuroinflammation is implicated in the onset, progression, and resolution of MS^6^. Microglia respond to pathological stimuli by producing inflammatory cytokines such as IL-1, IL-6, and TNF-α^70,72^ and by upregulating enzymes involved in reactive oxygen species production^73,74^.

As *V. ratti* MHL0042 treatment was found to reduce pro-inflammatory cytokine expression in the spinal cord (Fig. 4f), we hypothesized that *V. ratti* MHL0042 may directly inhibit microglial activation in the CNS, thereby suppressing EAE severity. To test this hypothesis, spinal cord tissues were immunostained with microglial activation markers, Iba1 and CD68, and analyzed using fluorescent microscopy. Iba1 is a well-established marker specifically expressed in microglia, particularly during activation^75^, whereas CD68 is a lysosomal protein commonly used to identify activated, phagocytic microglia^76^.

**Fig. 5:**
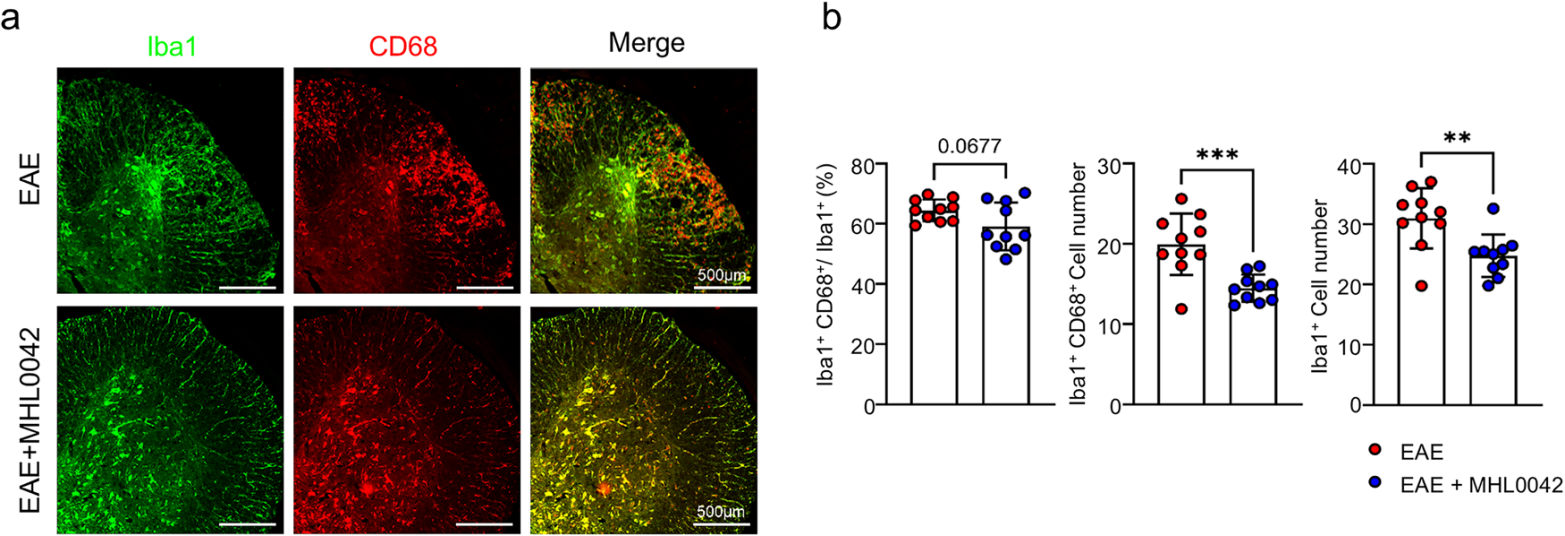
*Veillonella ratti* MHL0042 treatment suppresses microglia activation in experimental autoimmune encephalomyelitis mice. **a** Immunofluorescence staining of Iba1^+^ (green) and CD68^+^ (red) cells in the white matter of the spinal cord from experimental autoimmune encephalomyelitis (EAE) and EAE + *V. ratti* MHL0042 groups. Images are representative of 10 independent mice per group. **b** Quantification of the percentage of Iba^+^ CD68^+^ cells among total Iba^+^ cells, as well as absolute numbers of Iba^+^ CD68^+^ and Iba^+^ cells in EAE and EAE + *V. ratti* MHL0042 groups. Each dot represents the average of minimum three sections from an individual mouse. Data are presented as mean ± standard deviation from EAE (*n* = 10) and EAE + *V. ratti* MHL0042 (*n* = 10) groups. Statistical comparisons were performed using two-tailed unpaired *t* test. **P < 0.01; ***P < 0.001.

Our analysis revealed that the fluorescence intensity of Iba1^+^ and CD68^+^ cells (Fig. 5a), as well as the number of Iba1^+^CD68^+^ and total Iba1^+^ cells (Fig. 5b), was significantly reduced in the spinal cords of *V. ratti* MHL0042-treated EAE mice compared with that in untreated controls. Thus, *V. ratti* MHL0042 attenuates CNS inflammation and disease severity in EAE, at least in part, by suppressing microglia activation. Collectively, these findings suggest that gut microbiota modulation by *V. ratti* MHL0042 exerts systemic effects which extend to the CNS, where it suppresses microglia-driven neuroinflammation during EAE development.

### Integrated metabolomics and metagenome-based functional analyses link cecum microbiome remodeling to microbiota-derived metabolites following *V. ratti* MHL0042 treatment

Neuroinflammation suppression and microglial activation in the CNS following *V. ratti* MHL0042 administration, together with the absence of prominent inflammatory changes in intestinal tissues and bulk CD4^+^ T cell subset frequencies in the small intestinal lamina propria, prompted us to investigate whether microbiota-derived metabolites contribute to the gut–CNS axis. As our region-specific microbiota analysis identified the cecum as the primary site demonstrating the strongest separation between the EAE and EAE + *V. ratti* MHL0042 groups (Fig. 2) and revealed coordinated shifts in hub taxa which extended to distal compartments, we focussed our subsequent analyses on this compartment.

**Fig. 6:**
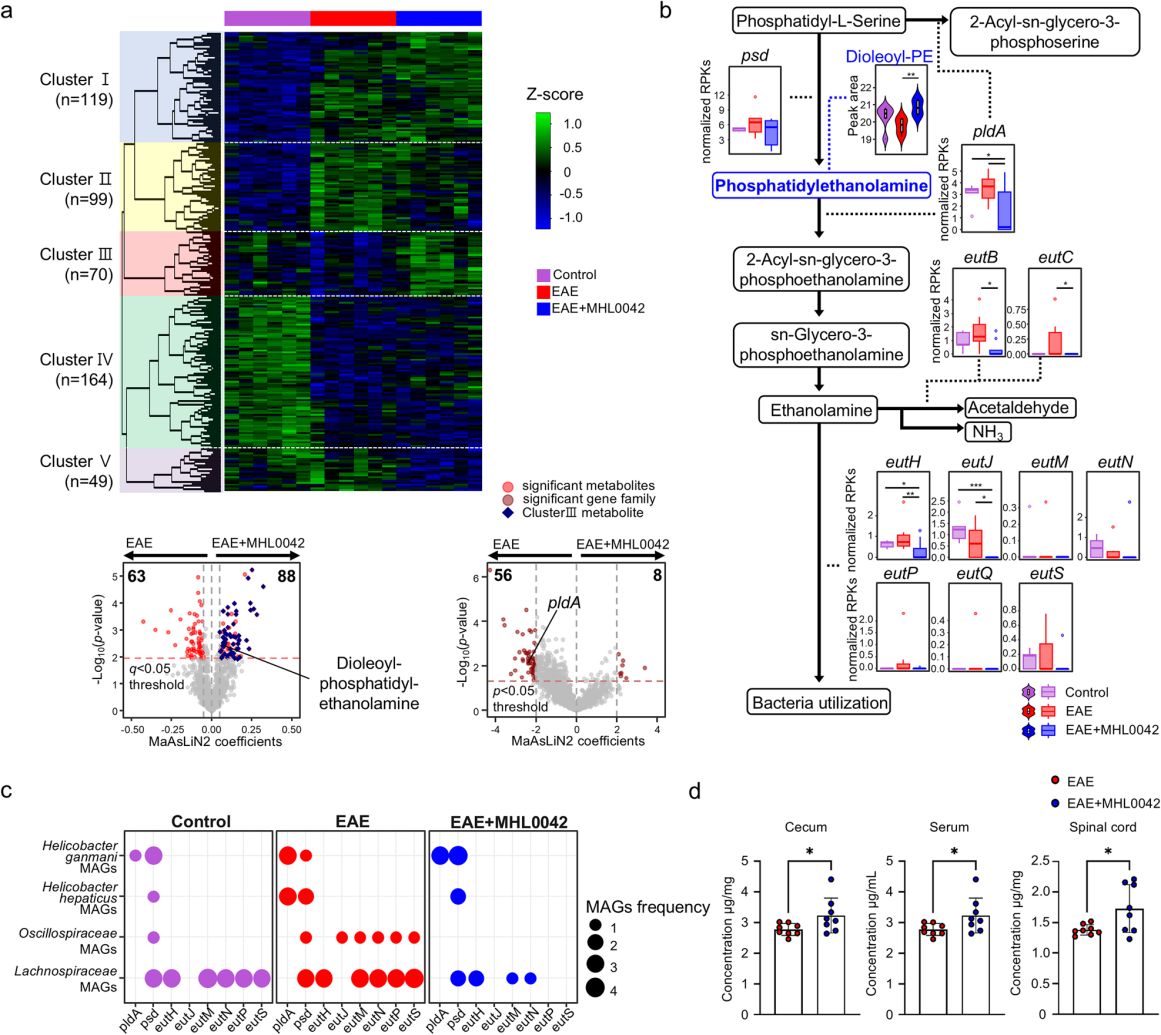
Comparison of cecal metabolites and associated microbial functional genes across groups. **a** Heatmap of significantly altered non-target metabolites among the treatment groups, with hierarchical clustering based on Spearman correlation. Volcano plots show differentially enriched metabolites and KEGG gene families between the experimental autoimmune encephalomyelitis (EAE) and EAE + *Veilonella ratti* MHL0042 groups. Significant features were identified using MaAsLiN2 (*q* < 0.05; |*ρ*| > 0.05 for metabolites, |*ρ* | > 2 for gene families). **b** Schematic representation of phosphatidylethanolamine biosynthesis pathways annotated with relevant gene families. *P*-values calculated using MaAsLiN2. *P < 0.05; **P < 0.01; ***P < 0.001. **c** Metagenome-assembled genomes were reconstructed from individual samples, and microbial contributors to phosphatidylethanolamine biosynthesis-related gene families were identified. Dot size represents the frequency of metagenome-assembled genomes harboring each gene family within each treatment group, linking microbial taxa to their associated functional genes. **d** Quantification of dioleoyl phosphatidylethanolamine in the cecum, serum, and spinal cord. Data are presented as mean ± standard deviation from EAE (*n* = 8) and EAE + *V. ratti* MHL0042 (*n* = 8) groups. Statistical comparisons were performed using unpaired Student's *t* test. *P < 0.05.

To determine whether cecal microbiota changes are accompanied by metabolite changes, we performed non-targeted metabolomic analysis of cecal contents. Principal component and hierarchical clustering analyses revealed distinct metabolic signatures among the control, EAE, and EAE + *V. ratti* MHL0042 groups (*q* < 0.05; Fig. 6a). Metabolites were clustered and enriched into the following five groups based on the abundance patterns: cluster I (119 metabolites) was enriched in both the EAE-induced groups; cluster II (99 metabolites) was enriched in the EAE group but reduced in the *V. ratti* MHL0042-treated group; cluster III (70 metabolites) was enriched in both the control and *V. ratti* MHL0042-treated groups; cluster IV (164 metabolites) was specific to the control group; and cluster V (49 metabolites) was enriched in both the control and EAE groups.

Volcano plot analysis (*q* < 0.05, coefficient > |0.05| in MaAsLiN2) identified 88 metabolites increased and 63 metabolites decreased in the *V. ratti* MHL0042-treated group compared with those in the EAE group; 61 candidate metabolites enriched in the *V. ratti* MHL0042 associated signature were prioritized for downstream integration with metagenomic functional profiles.

We next analyzed whether these metabolomic shifts could be linked to changes in microbial functional potential. Using WMS, we compared DEG families between the EAE and EAE + *V. ratti* MHL0042 groups (*P* < 0.05; coefficient > |2| in MaAsLiN2) and identified 56 decreased and 8 increased gene families in the *V. ratti* MHL0042-treated group. Integrated metabolite and gene-family profiles highlighted a coherent lipid-related axis: expression of the phospholipase gene, *pldA* (K01058), strongly co-varied with phosphatidylethanolamine (PE)-related metabolites, and PE was significantly elevated in the *V. ratti* MHL0042-treated group (Fig. 6b). Phosphatidyl-L-serine can be converted to 2-acyl-sn-glycero-3-phosphoserine by *pldA*, or it can be converted to PE by *psd* (Fig. 6b). Notably, *pldA* expression was significantly reduced in the EAE + *V. ratti* MHL0042 group (*P* < 0.05), whereas *psd* expression remained unchanged. In addition, the concentration of DOPE (a bioactive PE-related lipid) was significantly increased in this group (*P* < 0.05). Moreover, expressions of genes involved in PE degradation (*eutB*, *eutC*, *eutH*, and *eutJ*) were also significantly decreased (*P* < 0.05), consistent with a shift in microbiome functional potential that could favor DOPE accumulation in the *V. ratti* MHL0042-treated group. Furthermore, the phospholipase activity was higher in the cecal content of EAE mice compared with that in naive control and *V. ratti* MHL0042-treated mice in the presence of exogenous DOPE oral administration (Supplementary Fig. 6). This result suggests that the microbiome of EAE mice is more potent to degrade DOPE into its derivatives and newly shaped gut microbiota community by *V. ratti* MHL0042 intervention suppressed its breakdown.

To further identify microbial contributors to these changes, gene content differences were analyzed using MAGs reconstructed from short-fragment metagenome data (Fig. 6c). After quality-filtering and taxonomic assignment, the following four high-quality MAGs were identified as contributors to this pathway: Lachnospiraceae MAGs, Oscillospiraceae MAGs, *Helicobacter hepaticus* MAGs, and *Helicobacter ganmani* MAGs. The *psd* gene was detected across all four MAGs, whereas *pldA* was detected specifically in *Helicobacter* MAGs. By contrast, *eutH* and *eutJ* were detected in Lachnospiraceae and Oscillospiraceae MAGs, supporting a partitioning of PE/DOPE-related functional potential across distinct microbiome members. Importantly, the abundances of Lachnospiraceae, *H. hepaticus*, and *H. ganmani* positively correlated with the cecal network hub candidate taxa, UC_Oscillospiraceae_SGB43541, in previous analysis (Fig. 3). These associations suggest that the hub-centered microbiota remodeling observed following *V. ratti* MHL0042 treatment may influence which taxa dominate the microbiota, providing a mechanistic bridge between cecal network analysis and coordinated shifts in PE/DOPE-related functional potential and metabolite levels.

To investigate whether cecal DOPE accumulation reflected systemically and in the CNS as the target site, serum and cervical spinal cord *V. ratti* MHL0042 were collected for DOPE quantification using LC–MS. DOPE levels were higher upon *V. ratti* MHL0042 intervention than those in EAE control mice in both serum and spinal cord samples, mimicking the pattern observed in cecal content (Fig. 6d). Therefore, cecal DOPE alteration also extends systemically and to the CNS, thus allowing regulation of the CNS immune response.

In summary, *V. ratti* MHL0042 treatment is associated with cecum-centered microbiome remodeling, including coordinated changes in hub candidate taxa; this ecological shift co-occurs with distinct metabolomic signatures and MAG-resolved functional potential linked to PE/DOPE metabolism, which extends systemically and prolongs in the CNS. Together, these integrated results nominate PE and its bioactive form DOPE as candidate microbiota-derived mediators which may attenuate EAE severity via the gut–CNS axis modulation.

### Gut microbiota-derived metabolite DOPE suppresses EAE pathogenesis and microglial activation

Metabolomic profiling of *V. ratti* MHL0042-treated EAE mice revealed elevated DOPE levels, which corresponded with potentially decreased *pldA* expression in the gut microbiota (Fig. 6). Consequently, we hypothesized that DOPE functions as a bioactive metabolite mediating the protective effects of *V. ratti* MHL0042. To test this hypothesis, mice were administered DOPE intraperitoneally for 5 days before EAE induction. Daily administration was continued until the onset of clinical symptoms, followed by alternate-day injections throughout the disease course.

**Fig. 7:**
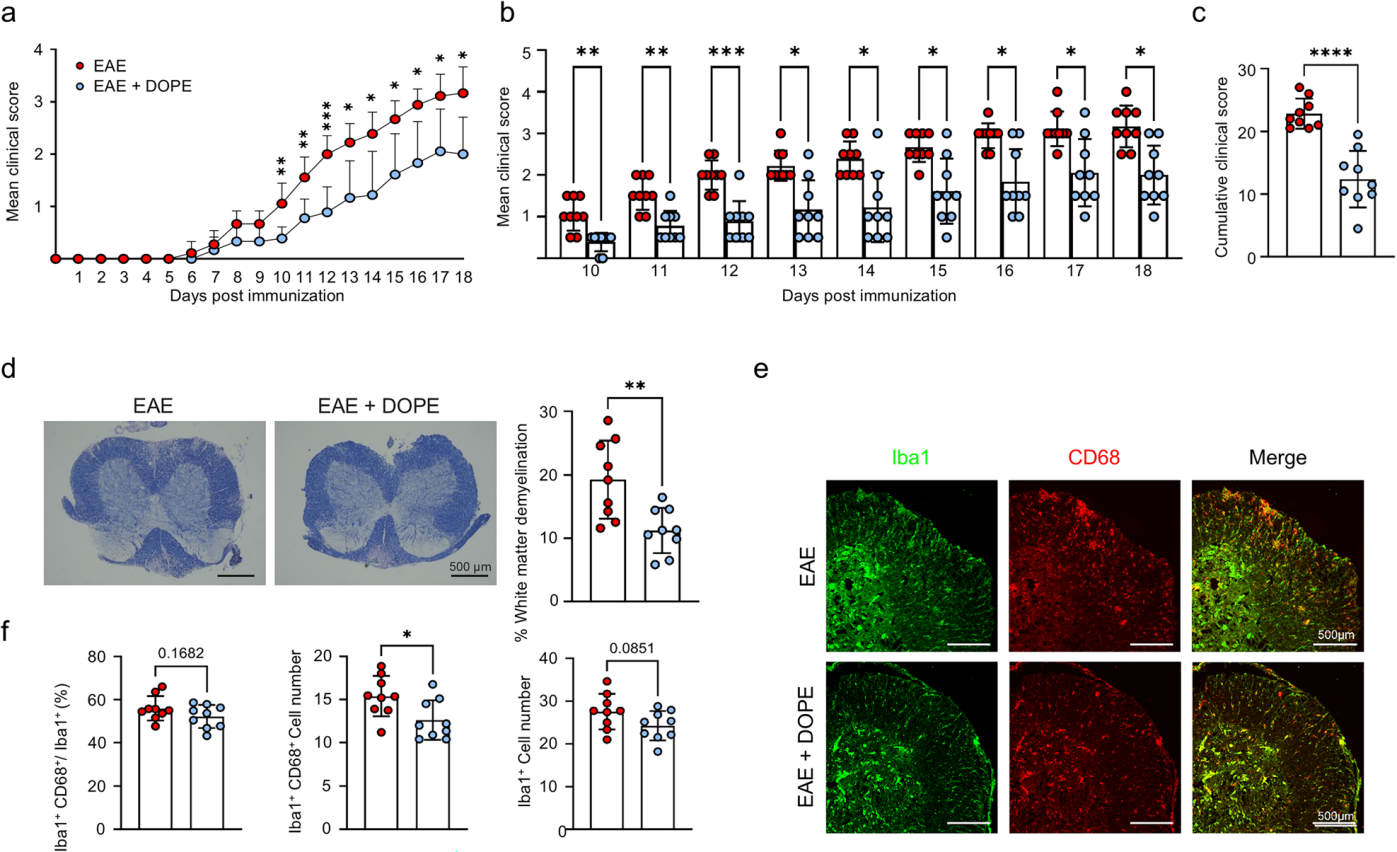
Amelioration of experimental autoimmune encephalomyelitis symptoms and suppression of microglia activation by dioleoyl phosphatidylethanolamine treatment. **a** Mean clinical score of experimental autoimmune encephalomyelitis (EAE) and EAE + DOPE groups. **b** Mean clinical score of EAE and EAE + DOPE groups showing individual mouse clinical score. **c** Cumulative clinical scores of EAE and EAE + DOPE groups. **d** Luxol fast blue staining of spinal cord sections and quantification of white matter demyelination in EAE and EAE + DOPE groups. **e** Immunofluorescence staining of Iba1^+^ (green) and CD68^+^ (red) cells in the white matter of the spinal cord from EAE and EAE + DOPE groups. Images are representative of 10 independent mice. **f** Quantification of the percentage of Iba^+^ CD68^+^ cells among total Iba^+^ cells, as well as absolute number of Iba^+^ CD68^+^ and Iba^+^ cells. Each dot represents the average of at least three sections from a single mouse. Data are presented as mean value ± standard deviation from EAE (*n* = 9) and EAE + DOPE (*n* = 9) groups. Data are representative of at least two independent experiments. Data were compared using two-tailed unpaired *t* test. *P < 0.05; **P < 0.01; ***P < 0.001; ****P < 0.0001. DOPE, dioleoyl phosphatidylethanolamine.

Compared with the control, DOPE treatment significantly reduced clinical EAE scores from disease onset through the experimental end point (Fig. 7a,b). This was further supported by a significantly lower cumulative clinical score in DOPE-treated mice (Fig. 7c), suggesting that systemic DOPE administration can attenuate EAE progression. Histological analysis revealed that untreated EAE mice exhibited extensive demyelination, particularly in the dorsal and ventral funiculus, characterized by the loss of myelin fibers and increased infiltration of nucleated immune cells. By contrast, DOPE-treated mice showed improved white matter integrity and reduced demyelination in these regions (Fig. 7d).

Considering that *V. ratti* MHL0042 suppresses EAE severity by modulating MOG-specific pathogenic CD4^+^ cells in the CNS and suppressing microglial activation, we next assessed whether DOPE exerts comparable immunomodulatory effects. The recruitment of MOG_35-55_-specific CD4^+^ cells in the spinal cord was diminished by DOPE treatment (Supplementary Fig. 7a) and IL-17A and IFN-γ production was reduced in the culture supernatants of MOG-re-stimulated spinal cord lymphocytes from DOPE-treated EAE mice (Supplementary Fig. 7b). Furthermore, immunofluorescence staining of spinal cord sections using Iba1 and CD68 as markers of phagosome-activated microglia revealed that DOPE administration significantly reduced the number of Iba1⁺CD68⁺ microglia, particularly in white matter areas, emphasizing the role of DOPE in suppressing microglia activation (Fig. 7e,f).

In summary, these findings identify DOPE as a functional gut microbiota-derived metabolite, the levels of which are elevated following *V. ratti* MHL0042 administration. DOPE attenuates EAE pathogenesis by suppressing pro-inflammatory cytokine production by pathogenic CD4^+^ T cells and inhibiting microglial activation in the CNS.

### DOPE inhibits the pro-inflammatory properties of activated microglia

**Fig. 8:**
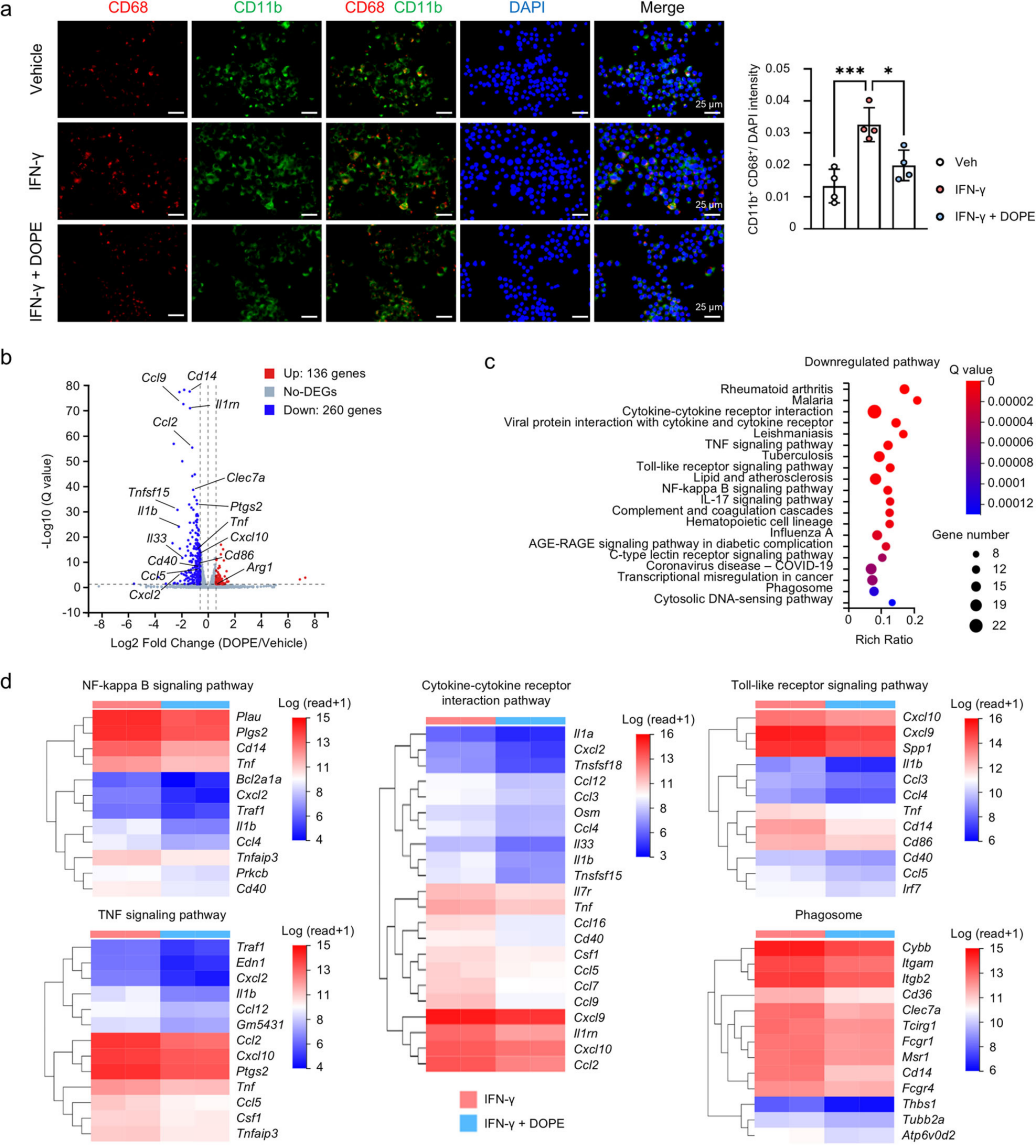
Inhibition of microglia activation and pro-inflammatory cytokine production by DOPE treatment in vitro. **a** Immunofluorescence staining of CD68^+^ (red) and CD11b^+^ (green) cells, counterstained with DAPI, in microglial (IMG) cells treated with vehicle or 25 μM DOPE in the presence or absence of 5 ng/ml interferon-γ (IFN-γ). Images are representative of four biological replicates (magnification, ×400). Quantification of CD11b^+^CD68^+^ mean fluorescence intensity normalized to DAPI is shown. Each dot represents the average of three different areas from the same well. Data are presented as mean ± standard deviation, representative of two independent experiments. Statistical comparisons were performed using one-way analysis of variance. *P < 0.05; ***P < 0.001. **b** Volcano plot of DEGs between IFN-γ-stimulated IMG cells treated with either vehicle or 25 μM DOPE. DEGs were identified using 1.5 log2 fold change cut-off (*q* < 0.05). **c** Downregulated KEGG enrichment analysis of differential gene set in DOPE-treated IFN-γ-induced IMG cells. **d** Heatmap of DEGs under specific pathways which are downregulated upon DOPE treatment in IFN-γ-induced IMG cells. Data are presented from two biological replicates. DAPI, 4′,6-diamidino-2-phenylindole; DEG, differentially expressed gene; DOPE, dioleoyl phosphatidylethanolamine; NF, nuclear factor; TNF, tumor necrosis factor; Veh, vehicle.

To validate the direct effects of DOPE on microglial activation, we used IFN-γ-stimulated IMG cells, to mimic the activation induced by T cell-derived cytokines observed in the EAE mouse model. Following DOPE treatment, cells were immunostained for CD68 and CD11b, which are markers associated with activated microglia. IFN-γ stimulation markedly increased the fluorescence intensity of CD11b⁺CD68⁺ cells, whereas co-treatment with DOPE significantly suppressed this signal (Fig. 8a), indicating that DOPE can directly inhibit IFN-γ-induced microglial activation.

This observation was further validated using flow cytometry. IFN-γ treatment significantly increased the proportion of CD11b⁺CD45^int^CD68⁺ cells, indicative of activated microglia (Supplementary Fig. 8a,b), and concurrently reduced the proportion of CD11b⁺CD45^int^CX3CR1⁺ cells, a population representative of homeostatic microglia. Notably, co-treatment with DOPE not only reduced the frequency of activated CD11b⁺CD45^int^CD68⁺ cells but also restored the proportion of homeostatic CD11b⁺CD45^int^CX3CR1⁺ microglia (Supplementary Fig. 8a,b). Thus, DOPE suppresses microglial activation and promotes a shift toward an anti-inflammatory phenotype.

To obtain deeper insights into gene expression alteration by DOPE, RNA-sequencing of IFN-γ-stimulated IMG cells treated with vehicle or DOPE was conducted. In total, 396 DEGs were identified, with 136 upregulated and 260 downregulated genes by DOPE treatment. Expressions of several pro-inflammatory chemokines (*Ccl2*, *Cxcl2*, *Ccl5*, and *Cxcl10*) and cytokines (*Il1b*, *Tnf*, and *Il33*) were reduced upon DOPE treatment (Fig. 8b). Gene set enrichment analysis using KEGG revealed the downregulation of pro-inflammatory pathways, including the cytokine–cytokine receptor interaction, TNF signaling, Toll-like receptor signaling, NF-kappa B signaling, and phagosome pathways (Fig. 8c,d). Particularly, DOPE reduced the expression of NF-κB target genes, including *Ptgs2*, *Il1b*, and *Bcl2a1a*, and NF-κB-dependent TNF response chemokines, including *Ccl2*, *Ccl5*, *Cxcl2*, and *Cxcl10* (Fig. 8d). Furthermore, DOPE treatment reduced the expression of *Cd40*, which is the upstream of NF-κB and is mediated by endogenous TNF-α and induced by IFN-γ^77^. These data suggest that DOPE suppresses inflammatory phenotypes in IFN-γ-stimulated IMG cells through the TNF–NF-κB pathway. DOPE also modulated amino acid metabolism, including arginine biosynthesis, tryptophan metabolism, biosynthesis of amino acids, and alanine, aspartate, and glutamate metabolism (Supplementary Fig. 8c).

Quantitative PCR was performed to assess alterations in pro-inflammatory cytokine and anti-inflammatory marker expressions. Consistent with these phenotypic changes, DOPE treatment significantly reduced the mRNA expression of pro-inflammatory mediators including *Il1b*, *Tnfa*, and *Nos2* in IFN-γ-stimulated IMG cells compared with those treated with IFN-γ alone (Supplementary Fig. 8d). Furthermore, DOPE upregulated the gene expressions of *Cx3cr1* and *Trem2*, which are associated with homeostatic microglia, even in the absence of inflammatory stimuli (Supplementary Fig. 8e), highlighting its intrinsic anti-inflammatory potential.

Collectively, these results indicate that DOPE directly modulates microglial activation by suppressing pro-inflammatory signaling and promoting a homeostatic phenotype. This mechanistic insight reinforces the role of DOPE, a microbiota-derived metabolite, in attenuating EAE pathogenesis.

## Discussion

The gut microbiome is a critical factor influencing susceptibility, progression, and immune responses in MS^11,78,79^. Although the precise contributions of individual bacterial taxa to MS patho-genesis are unclear, consistent alterations in gut microbiota composition have been observed in patients with MS^79^. In particular, abundance of the genus *Veillonella* notably decreases in patients with progressive MS compared with that in patients with relapsing–remitting MS, suggesting a potential protective role of this genus in modulating disease severity^28^. Furthermore, *Veillonella* contributes to host homeostasis by structuring the gut microbial ecosystem and producing bioactive metabolites^80,81^. Despite these associations, direct mechanistic evidence linking *Veillonella* to MS pathogenesis is limited.

Our study provides experimental validation that oral administration of *V. ratti* MHL0042 significantly ameliorates disease severity in EAE, a well-established murine MS model. These findings highlight the potential of *V. ratti* MHL0042 or microbiota-derived metabolites as promising therapeutics for modulating CNS-specific inflammation. Further research is needed to evaluate whether this effect is unique to *V. ratti* or shared across other *Veillonella* species.

Autoreactive CD4⁺ T lymphocytes, particularly T_H_1 and T_H_17 cells, are central to MS pathogenesis^82,83^. In passive EAE induction, involving an adoptive transfer of MOG-specific CD4^+^ T cells, we observed that *V. ratti* MHL0042 does not reprogram peripheral pathogenic CD4^+^ T cells to mediate its protective effect (data not shown). Moreover, systemic and mucosal frequencies of CD4⁺ T cell subsets (IL-17A⁺, IFN-γ⁺, and FOXP3⁺) were unaltered following *V. ratti* MHL0042 administration. However, a selective suppression of MOG-specific IFN-γ⁺ CD4⁺ T cells and their cytokine production were observed in the CNS, suggesting that *V. ratti* MHL0042 acts locally to modulate antigen-specific effector T cell responses in the CNS. A modest reduction in *Il17a* expression in the small intestine further indicates a limited but detectable effect on gut mucosal immunity. Notably, *V. ratti* MHL0042 did not alleviate inflammation in a dextran sulfate sodium-induced colitis model (data not shown). This finding underscores the context specificity of its immunomodulatory effects, favoring the gut–CNS axis rather than broader systemic or GI immune suppression.

Beyond T cell-mediated effects, microglia have a central role in both the initiation and resolution of neuroinflammation. In MS, activated microglia contribute to demyelination via antigen presentation and secretion of pro-inflammatory cytokines such as IL-12, IL-6, IL-1β, and TNF-α^5,7^. In our study, *V. ratti* MHL0042 treatment significantly reduced CNS expression of these cytokines and diminished microglial activation, as evidenced by reduced levels of Iba1 and CD68. CD68, a lysosomal marker associated with phagocytic activity, is typically upregulated in demyelinating lesions and is induced by IFN-γ^84^. Thus, the observed decrease in CD68 expression may result from a reduction in CNS-infiltrating T_H_1 cells and their associated cytokine output, thereby contributing to a less inflammatory and demyelinating CNS environment.

To investigate the potential mechanisms underlying these immunomodulatory effects, we analyzed gut microbiome and cecal metabolite profiles. The most prominent microbiota alterations between EAE and *V. ratti* MHL0042-treated EAE mice were observed in the cecum, and similar community shifts were also detected in the colon. Thus, *V. ratti* MHL0042 is associated with changes in microbial communities and their associated metabolites in both the cecum and colon, which may contribute to attenuating EAE severity.

Non-targeted metabolite profiles of cecal content revealed significant differences between the experimental groups. A comparison between the EAE and *V. ratti* MHL0042-treated EAE groups revealed that the levels of 88 metabolites were increased and those of 63 metabolites were decreased following treatment. Thus, *V. ratti* MHL0042 administration alters the gut microbiome such that it leads to distinct metabolic changes, highlighting the role of microbiome in modulating disease-associated metabolites. Notably, the microbial gene *pldA* may be associated with observed differences in metabolite profiles, and PE was identified as a potential metabolite linked to the attenuation of EAE severity through microbiome modulation. Further analysis of genes involved in PE metabolism revealed PE accumulation in the *V. ratti* MHL0042-treated group, potentially due to the reduced expression of PE-degrading enzyme genes such as *eutB*, *eutC*, *eutH*, and *eutJ*. In particular, DOPE levels were elevated in the *V. ratti* MHL0042-treated group. Thus, DOPE may act as a key bioactive molecule reducing EAE severity through microbiome-mediated mechanisms induced by *V. ratti* MHL0042 treatment.

DOPE, a PE species with two oleoyl (C18:1) chains, is known for its biophysical and signaling roles but has not previously been directly implicated in MS. Lipidomic studies have shown that PE levels decline during demyelination and are restored during remyelination, underscoring the critical role of membrane lipid composition in maintaining myelin integrity^85^. In our study, DOPE exhibited potential anti-inflammatory activity in microglia, reducing pro-inflammatory gene expression while upregulating homeostatic marker levels. DOPE suppressed IFN-γ-induced microglia putatively by inhibiting the TNF–NF-κB signaling pathway, supported by the downregulation of gene sets involved in both the TNF and NF-κB pathway. Currently, there is no direct evidence of DOPE or other PE species in modulating the NF-κB signaling pathway. However, phospholipase activation is essential for NF-kB activation by TNF-α^86^, suggesting phospholipid degradation or the product of phospholipase activity potentially inducing the activation of TNF-α-mediated NF-κB pathway. By contrast, other PE species with differing acyl chain compositions failed to elicit this dual response. Notably, certain PE derivatives, such as those containing C18:0 or C18:2 chains, can promote IFN-γ⁺ CD8⁺ T cell responses^87^, further highlighting the structural specificity of immunoregulatory potential of DOPE. Thus, DOPE functions as a bioactive microbial metabolite capable of attenuating microglial activation and neuroinflammation in EAE.

Although our findings in the EAE model are promising, their relevance to human MS remains to be determined. Publicly available human gut microbiome datasets for MS are still relatively limited and heterogeneous, and many WMS studies are insufficiently elaborated to robustly quantify specific genes or pathway-level features across individuals. In addition, most public MS WMS resources lack paired metabolomics, which precludes the direct assessment of DOPE and thereby limited rigorous validation of the proposed *pldA*/DOPE axis using existing observational cohorts. Moreover, our correlation-based network analyses are exploratory and do not support causal conclusions. Because networks inferred from compositional relative abundance data can be susceptible to spurious associations, the identified candidate hub taxa and inferred interaction changes should be interpreted as associations requiring functional validation. Furthermore, because DOPE was identified primarily through targeted metabolomics in the cecum, serum, and spinal cord of the EAE mouse model, it remains unclear whether DOPE levels are similarly altered in patients with MS or whether DOPE supplementation would exert comparable immunomodulatory effects in humans.

In summary, our study delineates a mechanistic link among *V. ratti* MHL0042, microbiota-derived metabolites, and CNS immune modulation. The therapeutic effects of *V. ratti* MHL0042 in an EAE model are potentially mediated through the targeted suppression of microglial activation and, to a lesser extent, via CNS-infiltrating effector T cells. These effects are potentially driven by accumulation of the gut microbiome-derived metabolite DOPE, whose structural specificity dictates its unique immunoregulatory functions. Our findings provide novel insights into gut–CNS communication and support the development of microbiome-based therapeutic strategies for MS. Further studies in human cohorts are needed to determine translational relevance and to assess whether *Veillonella*-derived metabolites can serve as viable targets for precision microbiome interventions in neuroinflammatory disorders.

## Supplementary information

Supplementary information

## Data Availability

The raw sequencing data obtained from this study are available in the EMBL SRA database under the study number accession number PRJEB88329 (http://www.ebi.ac.uk/ena/data/view/PRJEB88329).
