# Supplementary material for: Gut microbiome modulation by Veillonella ratti induces resistance to EAE pathogenesis via microbe-derived metabolites
Source: Exp Mol Med. 2026 Jul 14;58(7):2339–55. doi: 10.1038/s12276-026-01779-z (PMC13434020; doi:10.1038/s12276-026-01779-z)
Supplement: Supplementary file 1 [file 12276_2026_1779_MOESM1_ESM.pdf]

Supplementary Fig. 1

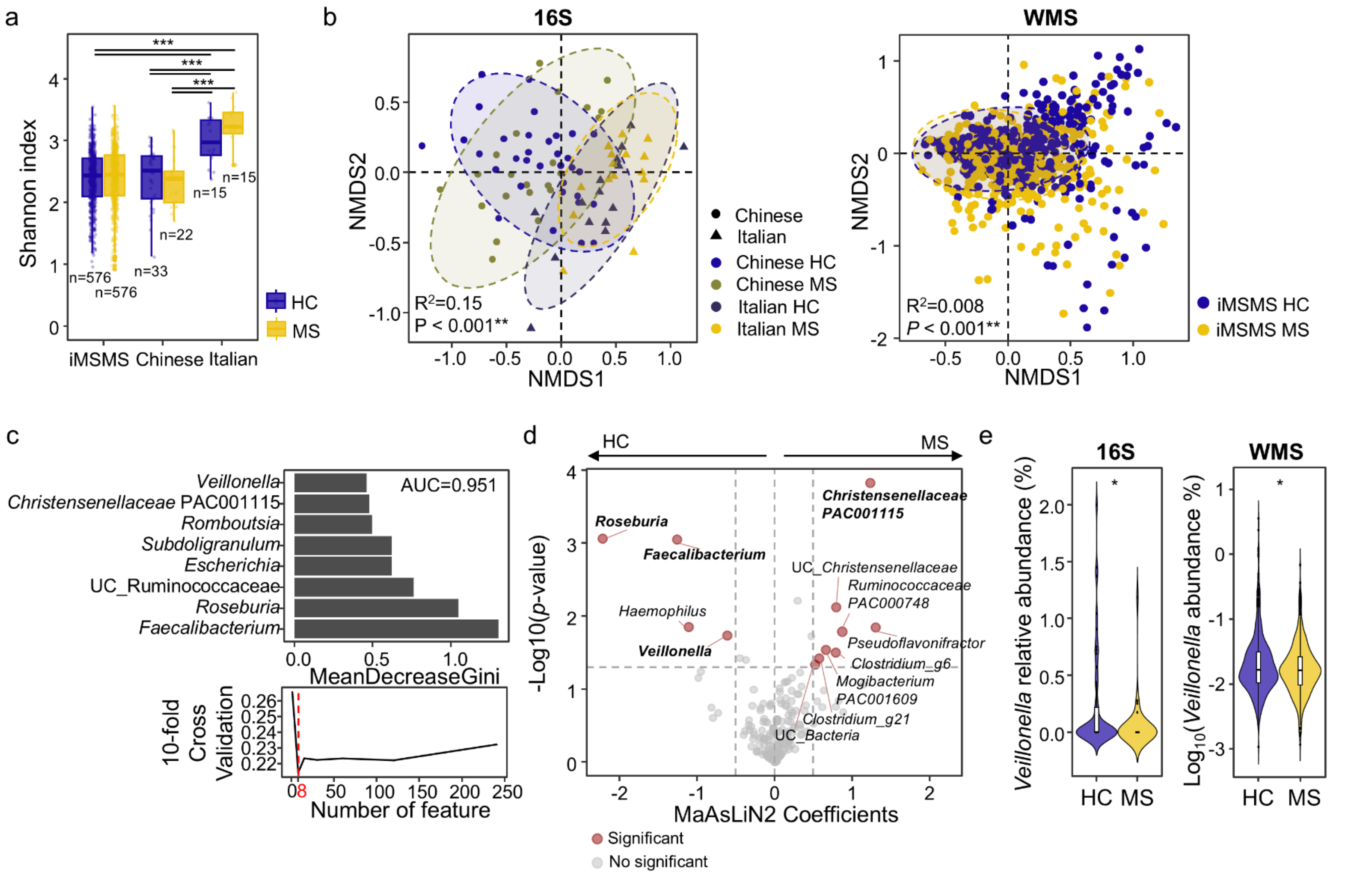

Supplementary Fig. 1. Difference in gut microbiota between healthy controls (HC) and multiple sclerosis (MS) patients, including reduced *Veillonella* abundance across cohorts. (a) Microbial diversity in each cohort were compared between HC and MS groups. Statistical significance was assessed using Dunn′s test with Benjamini-Hochberg false discovery rate (FDR) correction. $p < 0.001$; *** (b) Microbiota composition was visualized by non-metric multidimensional scaling (NMDS) based on Bray–Curtis dissimilarity, performed separately for 16S rRNA amplicon and whole-metagenome shotgun (WMS) datasets. *P* values were calculated using PERMANOVA. (c) Genus-level features ranked by importance in a Random Forest classifier distinguishing HC from MS. The eight most discriminative genera were selected based on 10-fold cross-validation. (d) Volcano plot of differentially abundant genera between HC and MS identified by MaAsLin2. The x-axis shows MaAsLin2 coefficients and the y-axis shows −log10(*P*-value). The significance threshold was set at |coefficient| > 0.5 and $P < 0.05$. (e) Relative abundance of *Veillonella* was compared between HC and MS patients, with analyses performed separately for the 16S rRNA amplicon and WMS datasets. Statistical significance was assessed using MaAsLiN2 while adjusting for cohort variation. $p < 0.05$; *.

Supplementary Fig. 2

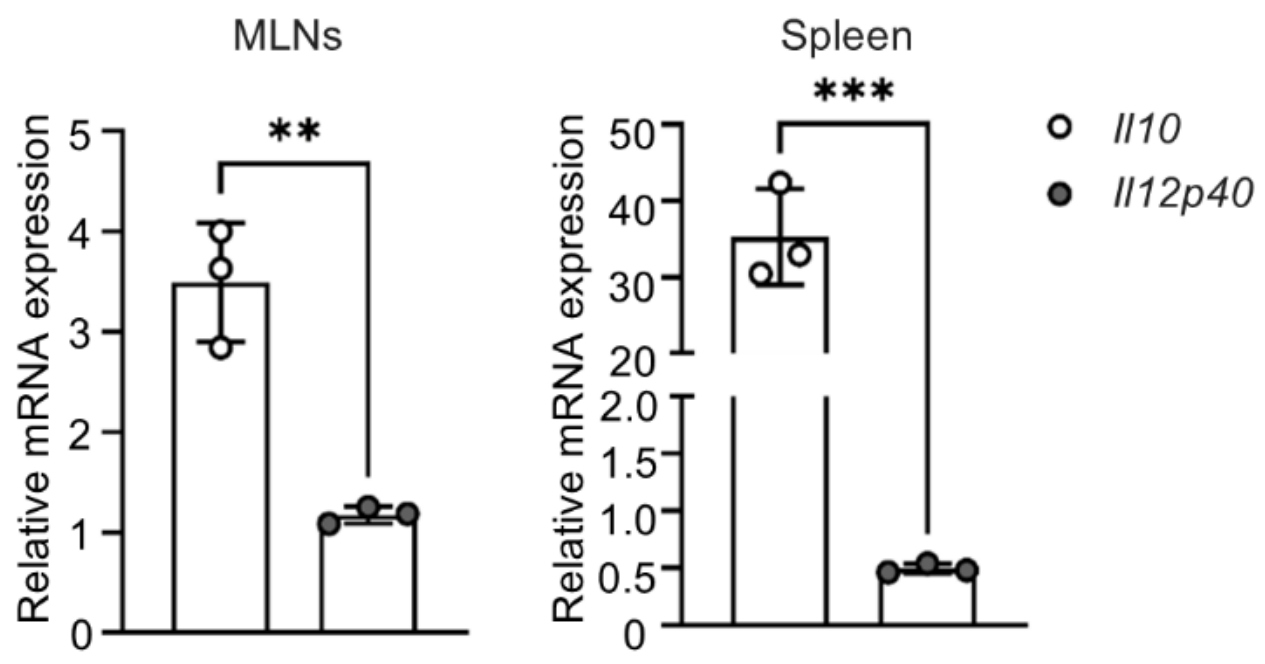

Supplementary Fig. 2. *Il10* and *Il12p40* expression in immune cells treated with *V. ratti* MHL0042. Inflammatory responses of immune cells cultured with or without *V. ratti* MHL0042 were assessed using RT-PCR. Relative mRNA expression levels of the anti-inflammatory cytokine, *Il10,* and proinflammatory cytokine, *Il12p40,* in mesenteric lymph node (MLN) and splenic cells treated with *V. ratti* MHL0042. Gene expression in cells cultured in the presence of *V. ratti* MHL0042 was normalized to that of cells cultured in its absence, with the latter designated as the reference value of 1 and results were shown as fold-change to this reference.Data are presented as mean ± standard deviation from three independent experiments. Statistical comparison was performed using Student's *t*-test, $p < 0.01$; **, $p < 0.001$; ***.

Supplementary Fig. 3

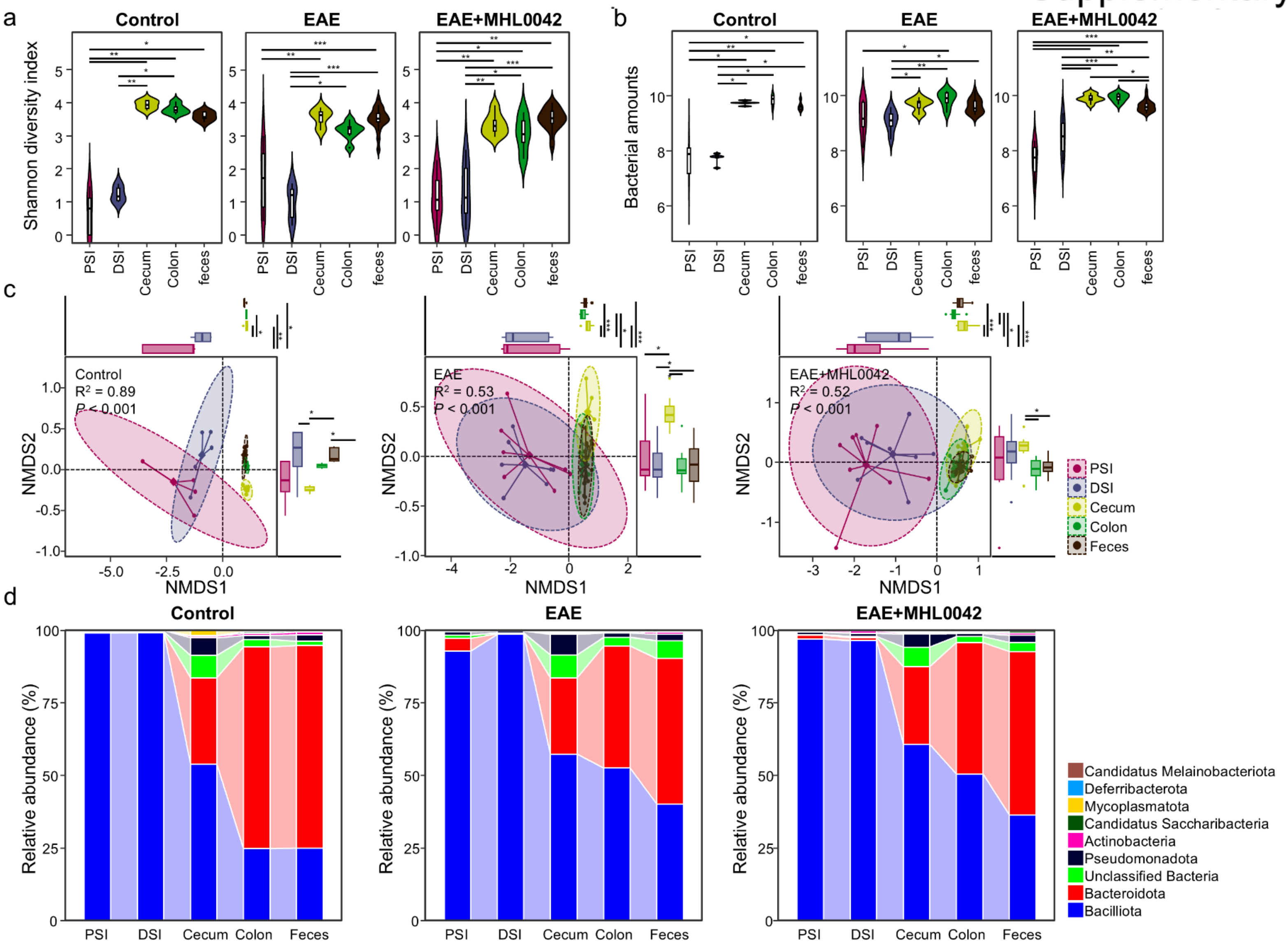

Supplementary Fig. 3. Comparison of the gut microbiota across different regions of the mouse gastrointestinal (GI) tract and treatment groups. (a) Microbial diversity and (b) total bacterial load were compared across GI tract regions. PSI, proximal small intestine; DSI, distal small intestine. Bacterial load was estimated using quantitative real-time PCR based on 16S rRNA gene copy number. Statistical significance was determined using Dunn's test with Benjamini-Hochberg false discovery rate (FDR) correction. (c) Microbiota composition was compared using non-metric multidimensional scaling (NMDS) based on Bray-Curtis dissimilarity. P values were calculated using PERMANOVA, and differences along the NMDS1 and NMDS2 axes were further evaluated using Dunn's test with FDR correction. (d) Relative abundances of dominant phyla were compared across GI tract regions. Bar plots represent the mean relative abundance in each region.

Supplementary Fig. 4

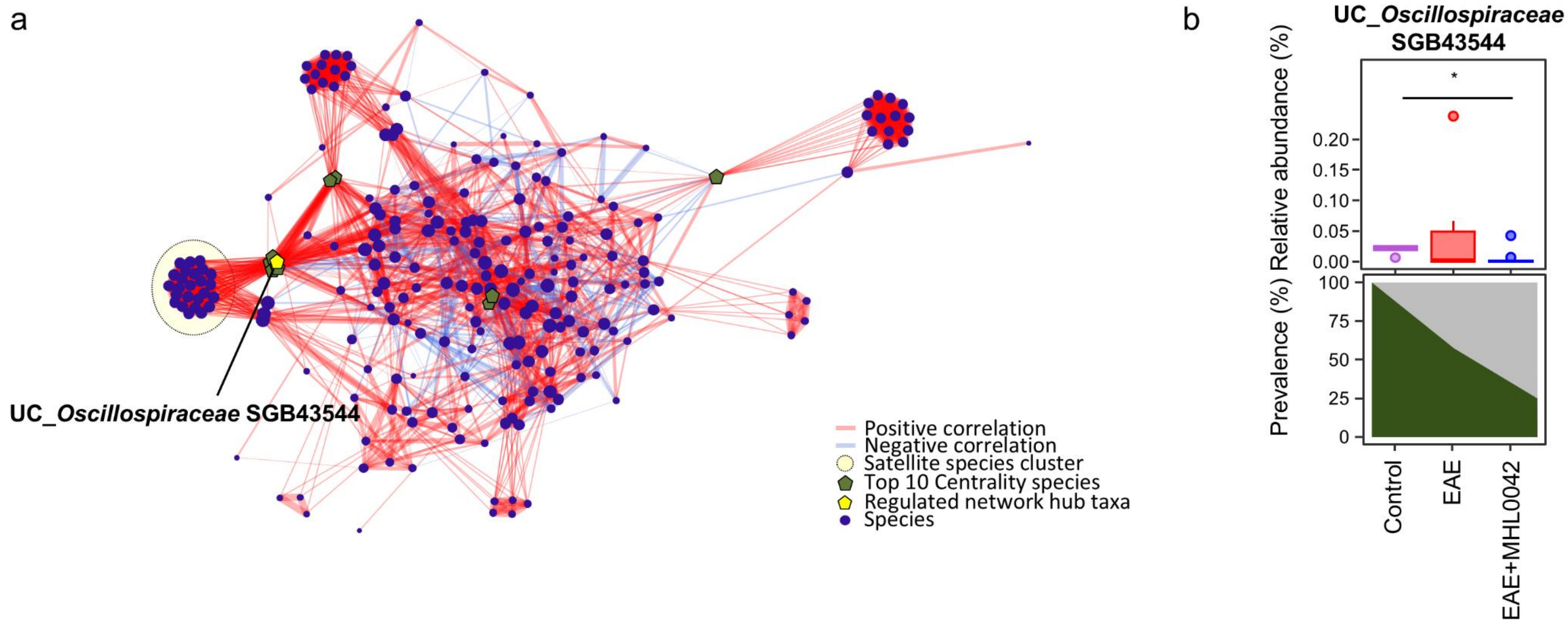

Supplementary Fig. 4. Cecum-associated network hub taxa remain associated with the colon microbiota in the EAE + *V. ratti* MHL0042 group. (a) Co-occurrence network of colon microbiota in the EAE + *V. ratti* MHL0042 group. Edges: significant Spearman correlations ($|\rho| > 0.7$, $p < 0.05$); red: positive, blue: negative; thickness: correlation strength. Node size: PageRank centrality. Green pentagons: top 10 central species; yellow: downregulated network hub taxa; dashed circles: associated satellite. (b) Relative abundance and prevalence of selected network hub taxa across groups. Significant differences were determined using MaAsLiN2. $p < 0.05$; *.

Supplementary Fig. 5

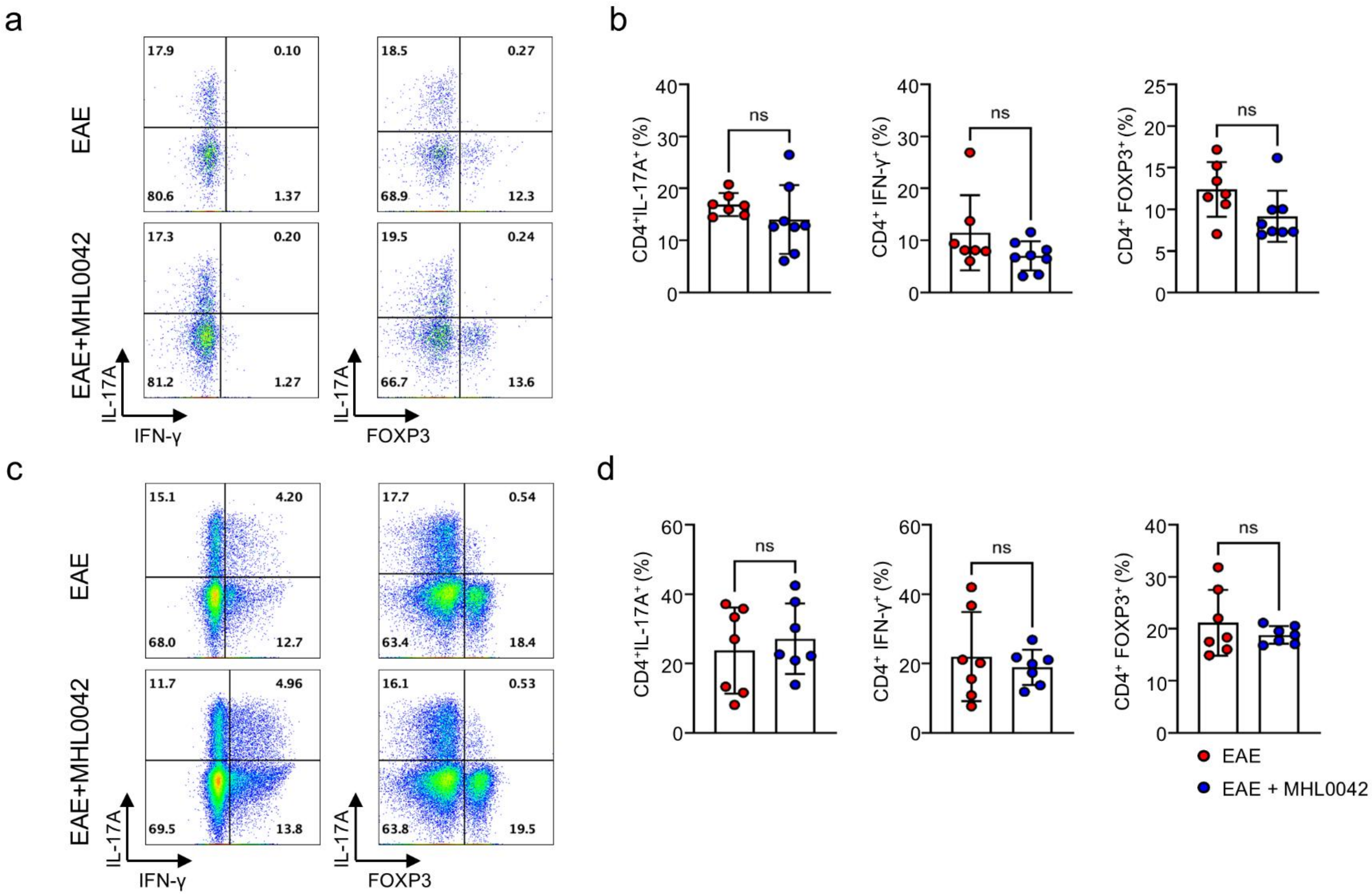

Supplementary Fig. 5. *V. ratti* MHL0042 does not directly suppress intestinal or CNS-resident CD4⁺ T cell inflammation. Total cells from the small intestinal lamina propria and spinal cord were isolated and analyzed using flow cytometry. Cells were gated under vital dye negative and CD4⁺ for further specific lineage analysis. (a, c) Representative FACS plots of CD4⁺ cells from small intestinal lamina propria (a) and spinal cord (c). (b, d) Percentages of specific lineages of CD4⁺, CD4⁺IL17A⁺, CD4⁺ IFN-γ⁺, and CD4⁺Foxp3⁺ cells, in the small intestinal lamina propria (b) and spinal cord (d). Data are presented as mean ± standard deviation.

Supplementary Fig. 6

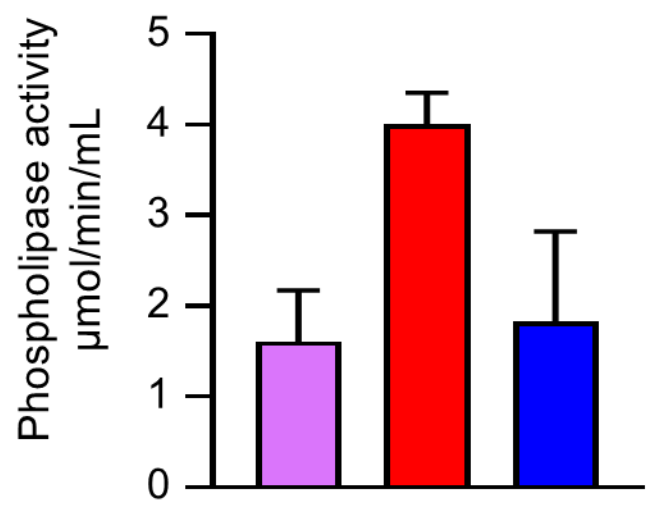

Supplementary Fig. 6. Alteration of phospholipase activity in EAE and *V. ratti* MHL0042-treated EAE mice. Phospholipase activity of cecal content derived from naïve control, EAE, and EAE + V. ratti MHL0042 groups in the presence of DOPE exogenous administration at day 14.

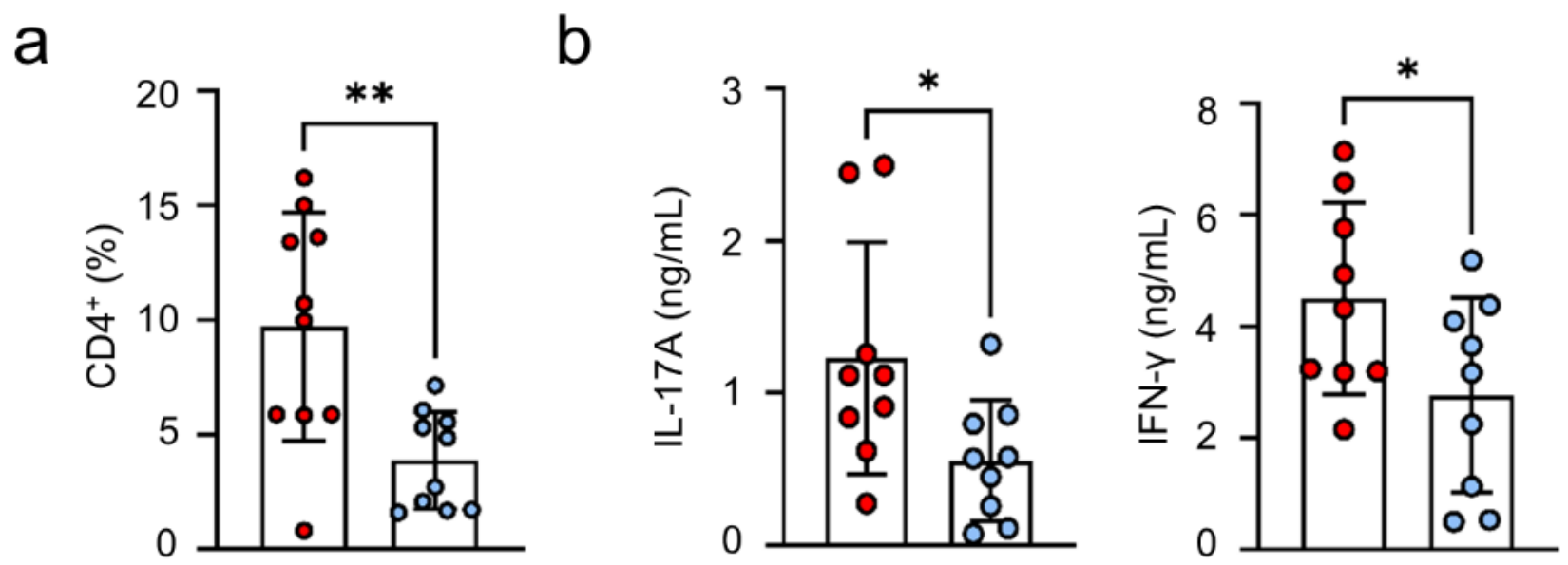

Supplementary Fig. 7. Reduction of recruited MOG_{35-55}-specific CD4+ cells and *ex vivo* cytokines production upon DOPE treatment. (a) The percentage of MOG_{35-55}-specific $CD4^+$ T cells isolated from the spinal cord of EAE mice and DOPE-treated EAE mice. (b) IL-17A and IFN-γ concentration in culture supernatants from $MOG_{35–55}$-restimulated spinal cord-infiltrating lymphocytes, measured by ELISA. Data are presented as mean ± standard deviation from three independent experiments. Statistical comparisons were performed using Student's *t*-test, $p < 0.05$; *, $p < 0.01$; **.

Supplementary Fig. 8

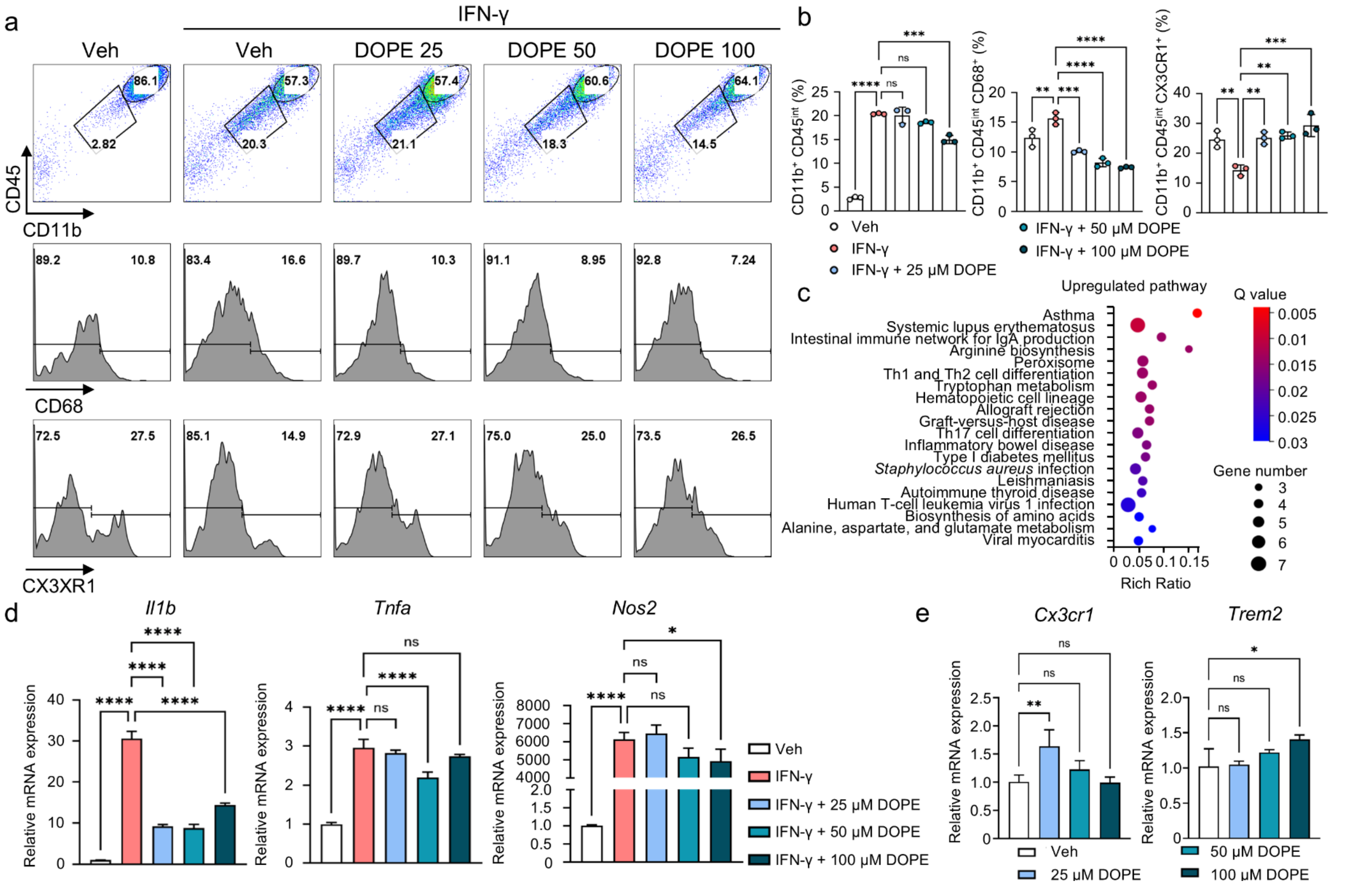

Supplementary Fig. 8. Inhibition of microglia activation and induction of homeostatic properties via DOPE treatment *in vitro*. (a) Representative FACS plots of IMG cells treated with vehicle or 25, 50, and 100 μM DOPE in the presence or absence of 5 ng/mL IFN-γ. CD11b⁺ CD45^int^ cells, histogram of CD68⁺ cells, and histogram of CX3CR1⁺ cells are shown. Images represent three biological replicates. (b) Percentages of CD11b⁺ CD45^int^, CD68⁺, and CX3CR1⁺ cells. Data are presented as mean ± standard deviation, representative of three independent experiments with three biological replicates per group. (c) Upregulated KEGG enrichment analysis of differential gene set in DOPE treated IFN-γ-induced IMG cells. (d) Relative mRNA expressions of of *Il1b, Tnfa,* and *Nos2* in IMG cells treated with vehicle or 25, 50, or 100 μM DOPE in the presence or absence of 5 ng/mL IFN-γ. (e) Relative mRNA expressions of *Cx3cr1* and *Trem2* in IMG cells treated with vehicle or with 25, 50, or 100 μM DOPE. The results were obtained using RT-PCR and normalized to β-actin expression. Data are presented as mean ± standard deviation from three independent experiments. Statistical comparisons were performed using one-way ANOVA, $p < 0.05$; *, $p < 0.01$; **, $p < 0.001$; ***, $p < 0.0001$; ****.

Supplementary information

Supplementary Table 1. List of primers used for RT-PCR analysis

Genes	Forward primer	Reverse primer
Nos2	CCA AGC CCT CAC CTA CTT CC	CTC TGA GGG CTG ACA CAA GG
Il6	TAG TCC TTC CTA CCC CAA TTT CC	TTG GTC CTT AGC CAC TCC TTC
Il1b	GCA ACT GTT CCT GAA CTC AAC	ATC TTT TGG GGT CCG TCA ACT
Tnfa	ACG GCA TGG ATC TCA AAG AC	GTG GGT GAG GAG CAC GTA GT
Il10	CCT CAG GAT GCG GCT GAG	GCT CCA CTG CCT TGC TCT TAT T
Il12p40	TGG TTT GCC ATC GTT TTG CTG	ACA GGT GAG GTT CAC TGT TTC T
b-actin	TTC GTT GCC GGT CCA CA	ACC AGC GCA GCG ATA TCG
Cx3cr1	CAA GCT CAC GAC TGC CTT CT	TGT CCG GTT GTT CAT GGA GTT
Trem2	ACC CTC TAG ATG ACC AAG ATG C	TTG GGC ACC CTC GAA ACT C

Supplementary Table 2. Top 10 central taxa based on PageRank scores in the cecum and colon microbiota networks.

Cecum			Colon		
Microbiota	PageRank score	Signi-ficance	Microbiota	PageRank score	Signi-ficance
UC_*Clostridiaceae* SGB43074	0.006865	b*	UC_Bacillota SGB41629	0.007687226	ab#
**UC_*Oscillospiraceae* SGB43541**	0.006793	bc*	**UC_*Oscillospiraceae* SGB43544**	0.007659181	b*
UC_ *Oscillospiraceae* SGB63209	0.006631	b*	UC_bacterium D16_29	0.007659181	b*c#
UC_Bacteria SGB41586	0.006625	a*	UC_*Oscillospiraceae* SGB43516	0.007659181	n.s.
UC_Bacillota SGB41629	0.006625	ab#	UC_*Oscillospiraceae* SGB43526	0.007659181	n.s.
Alistipes sp.	0.006427	ab#	UC_*Lachnospiraceae* bacterium_10_1	0.007659181	n.s.
**UC_ *Oscillospiraceae* SGB43544**	0.006335	bc*	Lactobacillus johnsonii	0.007585743	n.s.
UC_*Lachnospiraceae* SGB41535	0.006334	n.s.	UC_Bacillota SGB45230	0.006971365	n.s.
UC_Bacteroidales SGB40306	0.006194	n.s.	UC_bacterium_1XD42_1	0.006971365	n.s.
UC_Bacteroidota SGB40184	0.006155	b*	UC_Bacteria SGB41597	0.006788771	n.s.

*Statistical significance was determined using MaAsLiN2.
Comparisons: ‘a’; Healthy vs. EAE, ‘b’;Healthy vs. EAE + MHL0042, ‘c’; EAE vs. EAE + MHL0042.
Significance levels: $p < 0.05$; ‘*’, $q < 0.05$; ‘#’. $p > 0.05$; 'n.s.'
